# Structure–Activity Relationships and Biological Insights into PSMA-617 and Its Derivatives with Modified Lipophilic Linker Regions

**DOI:** 10.1021/acsomega.4c10142

**Published:** 2025-02-12

**Authors:** Martin Schäfer, Ulrike Bauder-Wüst, Mareike Roscher, Lucia Motlová, Zsófia Kutilová, Yvonne Remde, Karel D. Klika, Jürgen Graf, Cyril Bařinka, Martina Benešová-Schäfer

**Affiliations:** † Service Unit for Radiopharmaceuticals and Preclinical Studies, German Cancer Research Center (DKFZ), Im Neuenheimer Feld 280, 69120 Heidelberg, Germany; ‡ Research Group Translational Radiotheranostics, German Cancer Research Center (DKFZ), Im Neuenheimer Feld 280, 69120 Heidelberg, Germany; § Laboratory of Structural Biology, Institute of Biotechnology of the Czech Academy of Sciences, Průmyslová 595, 25250 Vestec, Czech Republic; ∥ Molecular Structure Analysis, German Cancer Research Center (DKFZ), Im Neuenheimer Feld 280, 69120 Heidelberg, Germany; ⊥ Nuclear Magnetic Resonance Laboratory, Institute of Organic Chemistry, Heidelberg University, Im Neuenheimer Feld 270, 69120 Heidelberg, Germany

## Abstract

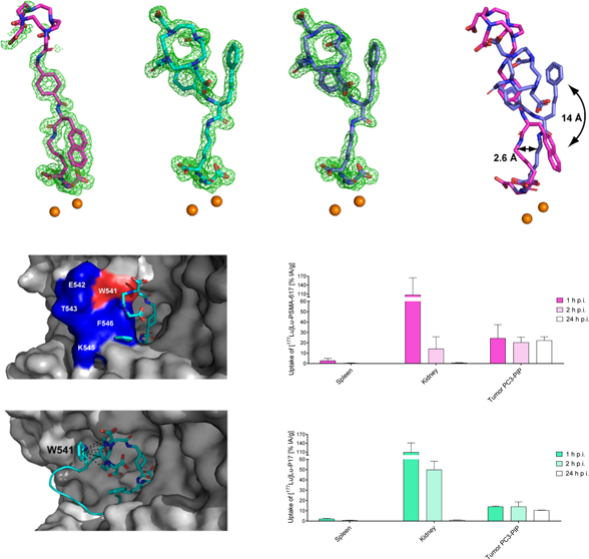

PSMA-617 is recognized as a benchmark ligand for prostate-specific membrane antigen (PSMA) owing to its broad utilization in prostate cancer (PCa) targeted radionuclide therapy. In this study, the structure–activity relationships (SAR) of PSMA-617 and two novel analogs featuring modified linkers were investigated. In compounds P17 and P18, the 2-naphthyl-l-Ala moiety was replaced with a less lipophilic 3-styryl-l-Ala moiety while the cyclohexyl ring in P18 was replaced with a phenyl group. The first ever crystal structure of the PSMA/PSMA-617 complex reported here revealed a folded conformation of the PSMA-617 linker while for the PSMA/P17 and PSMA/P18 complexes, the extended orientations of the linkers revealed linker flexibility within the PSMA cavity, a change in binding that can be exploited for the structure-guided design of PSMA-targeting agents. Despite structural differences from PSMA-617, the analogs maintained high PSMA inhibition potency, cellular binding, and internalization. In vivo biodistribution studies revealed comparable tumor uptake across all three compounds with P18 displaying higher spleen accumulation, likely due to phenyl ring lipophilicity. These SAR findings provide a strategic framework for the rational design of PSMA ligands, paving the way for the development of next-generation theranostic agents for PCa.

## Introduction

Cancer remains one of the leading causes of premature mortality worldwide, with 10.0 million deaths and 19.3 million new cases reported in 2020. This surge in cancer cases correlates with rising life expectancy, population growth, and shifts in socioeconomic development.^1^ Consequently, the development of improved diagnostic tools and targeted treatments has emerged as a key priority.

In prostate cancer (PCa), particularly metastatic castration-resistant prostate cancer (mCRPC), both diagnosis and therapy have advanced significantly with the development of prostate-specific membrane antigen (PSMA) ligands, which effectively target the active site in the molecule’s extracellular region.^2^ PSMA, a type II transmembrane protein, serves as a promising biomarker for targeted treatments, as it is highly expressed in poorly differentiated, metastatic, and hormone-refractory carcinomas, while showing minimal expression in healthy tissues.^3^ This selective expression helps to reduce side effects considerably. The effectiveness of PSMA-targeting inhibitors, typically featuring central Lys-urea-Glu or Glu-urea-Glu motifs and various linker structures, is underscored by the FDA and EMA approvals of [^177^Lu]Lu-PSMA-617 (Pluvicto, Novartis, Basel, Switzerland) for PCa treatment in March and December 2022, respectively.^4^

Despite the promising outcomes associated with PSMA-617 and a range of structurally diverse PSMA inhibitors demonstrating nanomolar binding affinity, the structure–activity relationships (SAR) of these ligands remain inadequately understood. Nevertheless, the linker structure has been identified as crucial for the biological activity of the overall scaffold, significantly influencing its pharmacological profile.^5−8^

**Figure 1:**
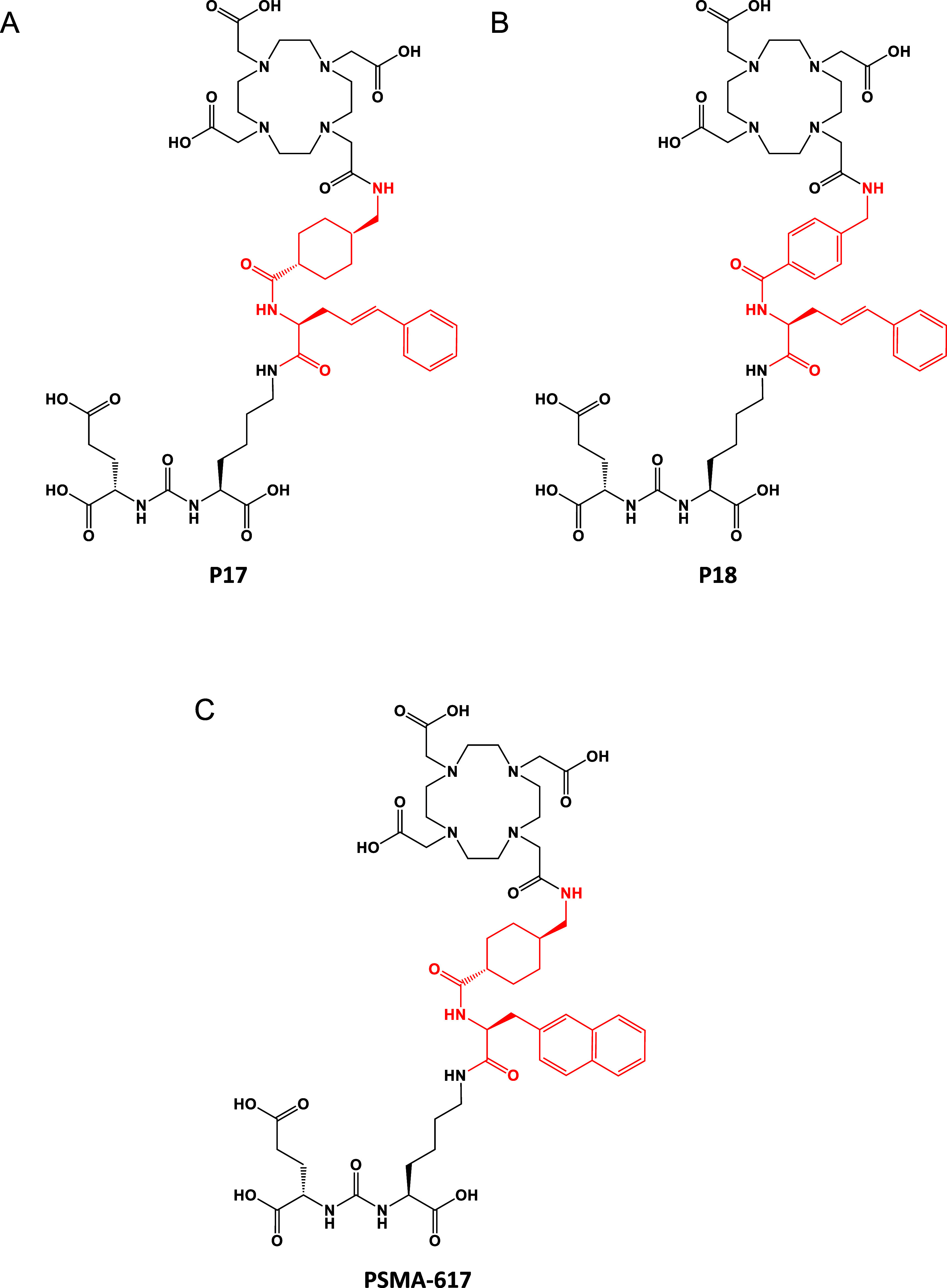
Structures of Glu-ureido-Lys-based DOTA-conjugated PSMA ligands synthesized in this work are presented as follows: (A) P17, which incorporates 3-styryl-l-Ala and *trans*-(aminomethyl)cyclohexyl moieties; (B) P18, which incorporates 3-styryl-l-Ala and 4-(aminomethyl)benzoic moieties; and (C) PSMA-617^6^, which incorporates 2-naphthyl-l-Ala and *trans*-(aminomethyl)cyclohexyl moieties in the linker region.

This study focuses on modifications to the linker region of the benchmark ligand PSMA-617.^6,9^ The SAR was conducted by replacing the strongly lipophilic aromatic 2-naphthyl moiety (2-naphthyl-l-Ala; log *K*_ow_ > 3) of PSMA-617 with a less lipophilic 3-styryl moiety (3-styryl-l-Ala; log *K*_ow_ < 3) (Figure 1). The primary objective was to demonstrate that the 3-styryl-l-Ala is a suitable alternative to 2-naphthyl-l-Ala while high affinity to PSMA is maintained. Secondary objectives included the exploration of (i) the redistributed lipophilicity (aromatic electron density) within the linker moiety and (ii) the reduced binding to serum albumin, which has frequently been noted for naphthyl moieties.^10^ In addition, the solid-phase synthesis technique was substantially refined through the optimization of reaction conditions and alternative reagent selection.

Both novel compounds, P17 and P18, were compared to PSMA-617 with respect to their structure and in vitro and in vivo biological characteristics. The replacement of the 2-naphthyl moiety in the linker region, which connects the PSMA binding motif with the DOTA chelator, was found not to have a major impact on PSMA binding affinity. Interestingly, both novel compounds displayed a distinct orientation of DOTA and the linker moiety in their interaction with PSMA, relative to PSMA-617. Based on these findings, the 3-styryl moiety is deemed suitable for incorporation into the linker structure and could serve as a promising alternative in the design of the next generation of PSMA ligands.

## Results

### Synthesis and Analytics

The 10-step solid-phase syntheses of P17 and P18 included several improvements compared to the originally established synthesis of PSMA-617.^5,6^ The preferred strategy offered various advantages for synthesizing the PSMA binding motif, including (i) successful scale-up from milligrams to grams, (ii) elimination of the hazardous triphosgene, (iii) the ability to activate both glutamate- and lysine-based building blocks, (iv) the replacement of the Alloc-protecting group with a more straightforward Fmoc-protecting group, and (v) a wider choice of solvents. A detailed description of the overall synthesis is provided in the Supporting Information (Schemes S1–S6).

The improved synthesis provided P17 and P18 in high purity (>99.9%) and with respective yields of 39% and 48% (Table 1), as verified by semipreparative high-performance liquid chromatography (HPLC). The purities of the compounds were evaluated using analytical HPLC (Figures S7–S9), while their identities were confirmed through both low-resolution electrospray ionization mass spectrometry (ESI-MS) (Figures S1–S3) and high-resolution matrix-assisted laser desorption/ionization MS (MALDI-MS) (Figures S4–S6). Further characterization was performed using nuclear magnetic resonance (NMR, vide infra).

**Table 1:** Analytical Data of Novel Ligands P17 and P18 as Well as PSMA-617

	linker region	molecular weight [g/mol]	retention time^a^ [min]	yield [mg (%)]	purity^a^ [%]
P17	3-styryl-l-Ala-TXA	1018.1	4.4	93 (39)	>99.9
P18	3-styryl-l-Ala-AMBA	1012.1	4.5	116 (48)	>99.9
PSMA-617	2-naphthyl-l-Ala-TXA	1042.2	4.7	140 (33)	>95.5

a Thermo Fisher Ultimate 3000 coupled to a variable wavelength detector and an Elysia-Raytest Gabi flow cell gamma detector; column: Chromolith C18 end-capped, 2 μm, 130 Å, 100 × 4.6 mm; eluents: (A) H_2_O + 0.1% trifluoroacetic acid (TFA), (B) acetonitrile (can) + 0.1% TFA; solvent gradient: 0–15 min 95–5% A; flow 2 mL/min; wavelength: 254 nm; temperature: room temperature (RT). The noncomplexed Lu-177 was observed at a retention time of 1.0 min.

### Structural Characterization by NMR

Compounds P17 and P18 were examined by ^1^H NMR and deemed to be pure and quite stable in DMSO solution with no sign of decomposition after several days. Characterization of the compounds was conducted extensively by one-dimensional (1D) ^1^H, ^13^C, and ^15^N NMR (Figures S10–S15) and two-dimensional (2D) spectra, viz. COSY, HSQC, and HMBC (Figures S16–S21). Previous assignments of analogous compounds^5^ were also used to provide unequivocal assignment of the ^1^H, ^13^C, and ^15^N signals except for the DOTA macrocyclic ring assignments which could not be effected with certitude due to dynamic effects causing severe line broadening and thereby precluding correlations in the 2D spectra pertinent to the DOTA ring. The dynamic effects arising from the slow motional averaging of the DOTA ring on the NMR time scale leading to severe broadening of the signals of the DOTA ring were alleviated for the ^13^C signals by the acquisition of spectra at lower field. Indeed, not all of the signals of the DOTA ring could be observed in the case of ^15^N, unequivocally assigned in the case of ^13^C, or uniquely discerned in the case of ^1^H. However, all other signals though were able to be fully assigned and the spectra were otherwise fully consistent with the proposed structures and for both P17 and P18 the assigned structures were confirmed in terms of proton count, carbon count, and carbon multiplicities.

### Determination of the Number of TFA Ions per Analyte Molecule

Quantification of the extent of protonation of the DOTA ring nitrogens was based on the number of TFA ions per analyte molecule and was evaluated using ^19^F and ^1^H NMR. For P17 and P18, an average of 2.37 and 2.36 TFA ions per analyte molecule, respectively, were measured.

### Inhibitor Potency In Vitro

The potency of the studied inhibitors against recombinant human PSMA was determined in vitro using an HPLC-based assay with fluorescein−γ-Glu–Glu as the substrate.^11^ The resulting calculated half-maximal inhibitory concentration (IC_50_) values were 0.05, 0.30, and 0.45 nM for PSMA-617, P17, and P18, respectively (Table 2). The subnanomolar inhibitory potencies were virtually identical for parent PSMA-617 and its P17/P18 derivatives, reflecting tight binding to PSMA via an extensive network of intermolecular contacts as observed in X-ray structures of PSMA/inhibitor complexes (see below).

**Table 2:** IC_50_ Values^a^ Determined via PSMA Enzyme and PSMA Cell-Based Assay

	PSMA-617 [nM]	P17 [nM]	P18 [nM]
PSMA	0.05 ± 0.03	0.30 ± 0.04	0.45 ± 0.09
LNCaP cell line	6.9 ± 2.2	12.9 ± 0.4	11.2 ± 4.8
C4-2 cell line	4.9 ± 0.9	15.5 ± 2.5	9.5 ± 3.0
PC3-PIP cell line	71 ± 8	150 ± 30	111 ± 9

a Data are average ± standard deviation (SD) (*n* = 3).

### Radiolabeling and Quality Control

Radiolabeling efficiency exceeded 96.5% for all three PSMA ligands (Figures S22–S24). Consequently, the resulting [^177^Lu]Lu-PSMA-617, [^177^Lu]Lu-P17, and [^177^Lu]Lu-P18 were utilized for subsequent in vitro and in vivo experiments without additional purification.

### *n*-Octanol/PBS Distribution Coefficients

All three radiolabeled compounds exhibited hydrophilic properties, as indicated by their negative log *D* values. [^177^Lu]Lu-P17 and [^177^Lu]Lu-P18 showed log *D* values of −3.36 ± 0.05 and −3.35 ± 0.04, respectively. [^177^Lu]Lu-PSMA-617 had a slightly higher log *D* of −3.27 ± 0.05, although this difference was not statistically significant.

### Cell-Binding Affinity Assay

To validate the IC_50_ values determined by the in vitro enzymatic assay, the PSMA-expressing cell line LNCaP, its daughter cell line C4-2, and the PSMA-overexpressing cell line PC-3 PIP were utilized to compare the cell-binding affinities of P17 and P18 to PSMA-617. The IC_50_ values of P17 and P18 were assessed by competitive binding against [^177^Lu]Lu-PSMA-617. PSMA-617 was found to bind to PSMA-positive LNCaP and C4-2 cell lines in the low nanomolar range with values of about 5 nM. In comparison, P17 and P18 were found to bind with values of about 15 and 10 nM, respectively (Table 2). For the transfected cell line PC-3 PIP, the IC_50_ values for all three compounds were determined to be approximately 10-fold higher. Overall, it was concluded that the inhibition potencies are essentially comparable for all three compounds within each specific cell line.

### Cellular Internalization Assay

To further characterize P17 and P18, internalization assays were conducted to determine whether the structural modifications of the linker have an impact on the uptake of the inhibitor–enzyme complex into the cell, which is critical for efficient retention in vivo. PC-3 PIP cells were incubated with [^177^Lu]Lu-PSMA-617, [^177^Lu]Lu-P17, and [^177^Lu]Lu-P18 and the surface-bound fractions were determined to be 26.8 ± 14.2, 20.1 ± 9.8, and 33.7 ± 12.8% of total applied activity (% AA), respectively (Table 3). Similar values were also observed for the internalized fractions of [^177^Lu]Lu-P17 and [^177^Lu]Lu-P18 in comparison to [^177^Lu]Lu-PSMA-617 (8.5 ± 2.4%, 8.7 ± 2.9%, and 8.2 ± 2.4% AA, respectively), indicating that the structural modifications do not influence the binding potential or uptake of P17 and P18 into the cells.

**Table 3:** Internalization Rates^a^ Determined via PSMA Cell-Based Assay Using PC3-PIP Cells

	[^177^Lu]Lu-PSMA-617 [ % AA]	[^177^Lu]Lu-P17 [ % AA]	[^177^Lu]Lu-P18 [ % AA]
total binding	26.8 ± 14.2	20.1 ± 9.8	33.7 ± 12.8
internalized	8.2 ± 2.4	8.5 ± 2.4	8.7 ± 2.9

a Data are average ± SD (*n* = 3).

### X-ray Structural Characterization of PSMA–Inhibitor Complexes

**Figure 2:**
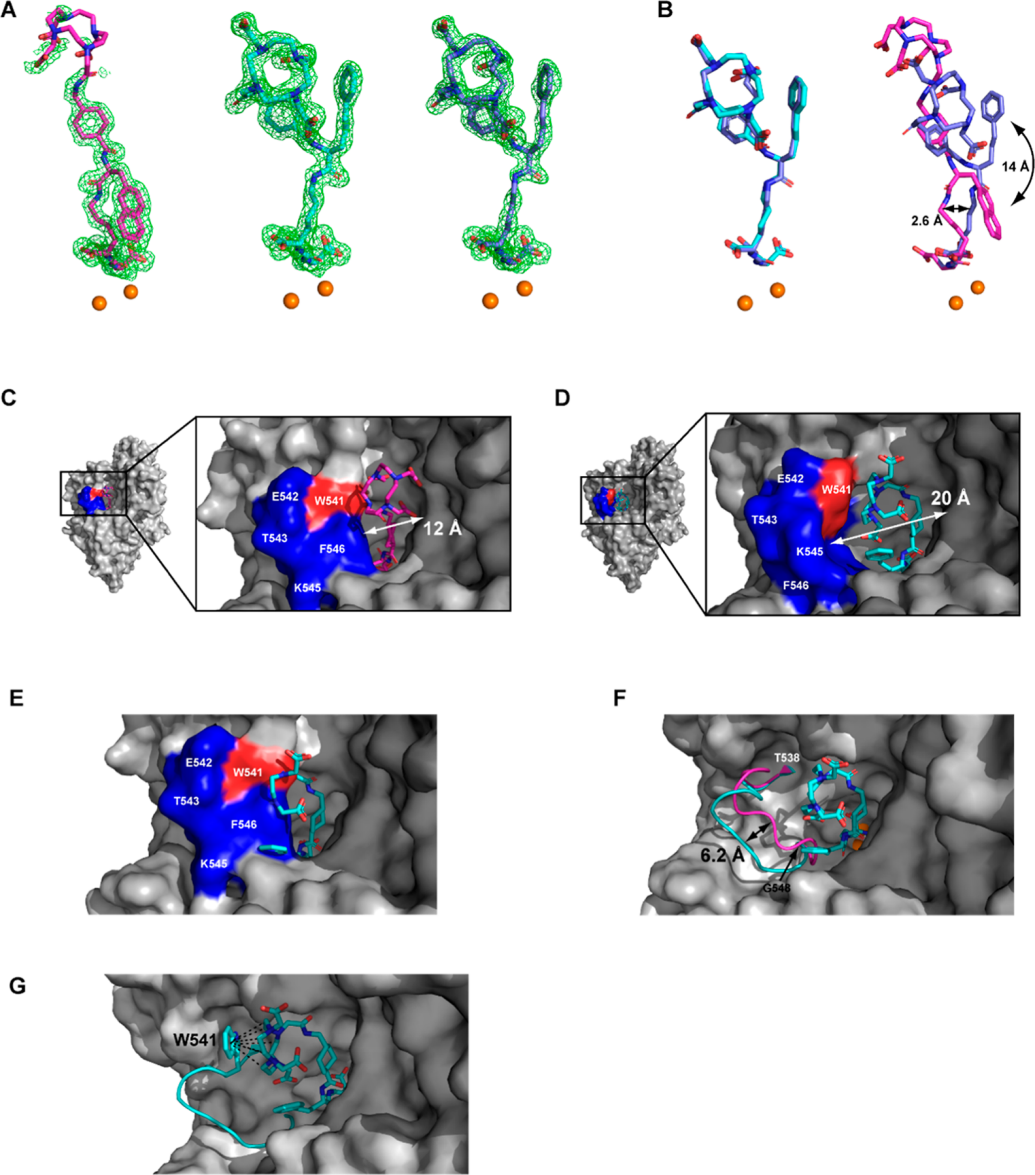
Structural characterization of PSMA–inhibitor complexes. (A) The |Fo| – |Fc| omit maps contoured at 3.0 σ are shown in green for the PSMA-617 (left, carbon atoms colored magenta), P17 (middle, carbon atoms colored cyan), and P18 (right, carbon atoms colored tv-blue) PSMA–inhibitor complexes. The active-site zinc ions are shown as orange spheres and inhibitors are in stick representation. Note the absent electron density for the DOTA moiety of PSMA-617 implying its positional flexibility due to a lack of interactions with PSMA. (B) Superposition of PSMA-617, P17, and P18 inhibitors extracted from corresponding PSMA–inhibitor complexes. While the atom positions of P17 and P18 are virtually identical (left), there are marked differences in the atom positions of the linker and the DOTA moieties of P18 and PSMA-617 (right). (C,D) Flexibility of the entrance lid of PSMA in PSMA–inhibitor complexes. PSMA is shown in surface representation (gray) with residues forming the entrance lid (Thr538–Gly548) colored blue and Trp541 colored red. For the PSMA–PSMA-617 complex (C), the lid is in a partially closed conformation, limiting the diameter of the entrance funnel to about 12 Å, while the open lid conformation in the PSMA-P17 complex (D) increases the diameter of the entrance funnel to about 20 Å. (E,F) Flexibility of the entrance lid is required to accommodate inhibitors with different distal moieties. PSMA–P17 and PSMA–PSMA-617 complexes were superpositioned on the corresponding Cα atoms. The putative binding of P17 (stick representation) to the PSMA protein extracted from the PSMA–PSMA-617 complex (surface representation). Note the steric clash between the inhibitor and the enzyme (P17 is partially obscured by the surface contour of the entrance lid indicating the necessity for lid repositioning for P17 binding (E). (F) The entrance lid as cartoon colored cyan and magenta for PSMA-P17 and PSMA-617, respectively. Notice differences in the lid conformation, where repositioning by up to 6 Å is required to accommodate P17. (G) The DOTA moiety of P17 engages the side chain of Trp541 of the entrance lid (cartoon representation) via an array of CH–π bonds indicated by broken lines.

To elucidate the patterns governing the interactions between inhibitors and PSMA, crystal structures of PSMA-inhibitor complexes were determined to the resolution limits of 1.58, 1.55 and 1.55 Å for PSMA-617, P17 and P18, respectively. As expected, the glutarate-urea docking module interacts with residues lining the S1′ pocket and the active site of PSMA in a manner consistent to other urea-based inhibitors described previously.^12,13^ The lysine P1 carboxylate function engaged the side chains of Asn519, Arg534, and Arg536, as observed for analogous PSMA–urea complexes.^12,13^ Interestingly, marked differences in atomic positions were observed for the DOTA moiety and the flexible linker (Figure 2A,B), along with interacting PSMA residues (Figure 2C,D,F).

In the PSMA–PSMA-617 complex, the wide diameter and partial flexibility of the PSMA entrance funnel allowed the 2-naphthyl function to fold inward, pushing the aminohexanoyl group toward the opposite wall of the funnel, thus effectively filling its lower part (Figure 2C). On the other hand, the corresponding less lipophilic 3-styryl moiety of P17 and P18 was oriented toward the exterior of the protein (Figure 2D), with its phenyl ring sandwiched between the Lys207 side chain (ε-NH_2_...ring center, 3.9 Å) and the DOTA moiety (4.0 Å). The differential atomic positioning of the distal parts of the inhibitors was facilitated by the structural plasticity of the “entrance lid” (Figure 2C,D,F), the flexible amino acid segment comprising residues Thr538–Gly548 of PSMA.^12,14^ In the case of the PSMA–PSMA-617 complex, the lid adopted a semiclosed conformation (Figure 2C), with the linker moieties following its contours. The 2-naphthyl group engaged in CH–π interactions with the Cα carbons of Ser547 (4.5 Å) and Gly548 (3.4 Å), while the adjacent cyclohexyl ring (in a chair conformation) was tightly packed against the phenyl ring of Phe546, with a distance of 5.1 Å between the ring centers. In the PSMA–P17 and PSMA–P18 complexes, the lid was pushed away from the center of the entrance funnel (up to 6.2 Å from the Cα carbons of Phe546), thus accommodating a less compact conformation of the inhibitor linker (Figure 2D–F).

Finally, electron density peaks in the |Fo| – |Fc| map were not visible for the DOTA moiety of PSMA-617 (Figure 2A), indicating an absence of intermolecular interactions with PSMA and suggesting its flexibility outside the PSMA entrance funnel. In contrast, the DOTA moiety was well resolved in the |Fo| – |Fc| maps of PSMA–P17 and PSMA–P18 complexes (Figure 2A), where its position was stabilized by a series of CH–π interactions between the methylene groups of the 12-membered tetraazacyclododecane ring and the indole group of the Trp541 side chain of the entrance lid (Figure 2G).

### Biodistribution Studies

**Figure 3:**
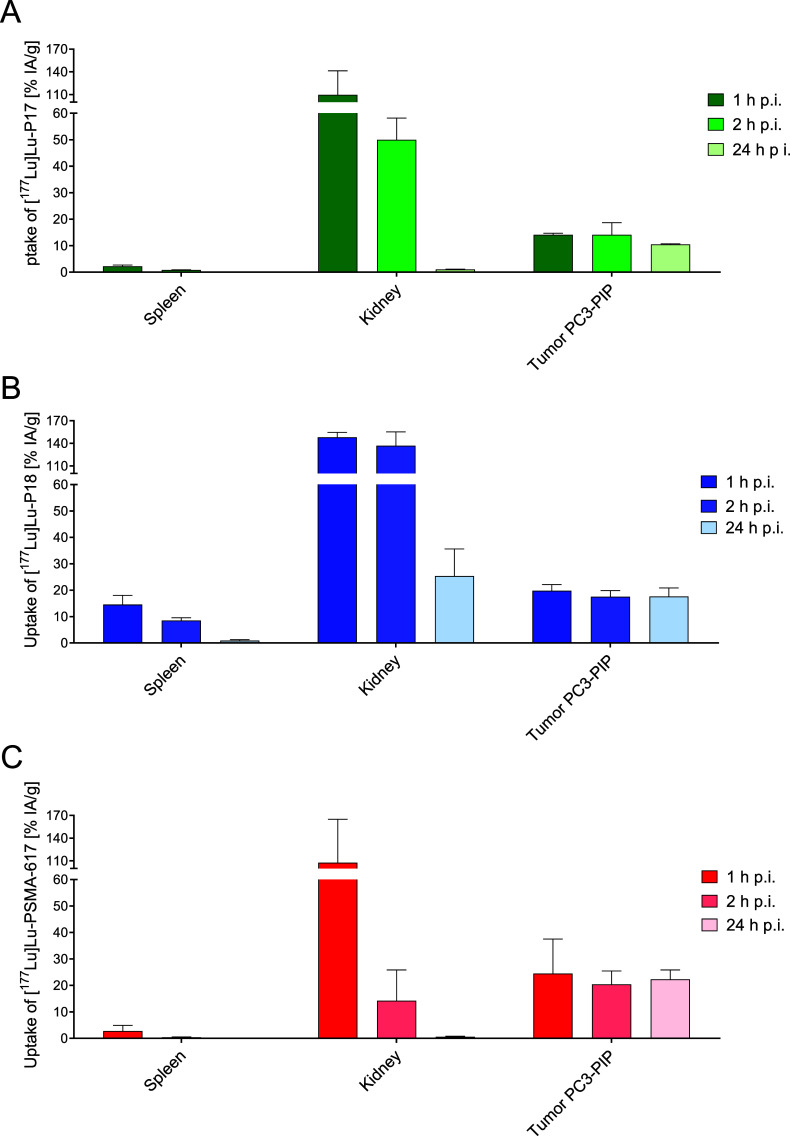
Uptake of radiolabeled PSMA ligands in the spleen, kidney, and PC3-PIP tumor at 1, 2, and 24 h p.i. is presented for (A) [^177^Lu]Lu-P17, (B) [^177^Lu]Lu-P18, and (C) [^177^Lu]Lu-PSMA-617. Data are average ± SD % IA/g (*n* = 3).

To assess the tissue distribution profile of the radiolabeled compounds, PC-3 PIP and PC-3 flu tumor-bearing BALB/c nu/nu mice were injected with [^177^Lu]Lu-P17, [^177^Lu]Lu-P18, and [^177^Lu]Lu-PSMA-617. The mice were sacrificed at 1, 2, and 24 h postinjection (p.i.) and their tumors, organs, and other relevant tissues were analyzed (Figure 3, Tables 4–6; Figures S25, S26, and Table S1). Similar to [^177^Lu]Lu-PSMA-617, both [^177^Lu]Lu-P17 and [^177^Lu]Lu-P18 exhibited a specific tumor uptake of 14.1 ± 0.6 and 19.8 ± 2.3% injected activity (IA)/g, respectively, at 1 h p.i., compared to 24.5 ± 13.0% IA/g for [^177^Lu]Lu-PSMA-617. After 24 h, the PC-3 PIP tumor retention of [^177^Lu]Lu-P17 and [^177^Lu]Lu-P18 decreased minimally to 10.5 ± 0.3% and 17.7 ± 3.2% IA/g, respectively, while [^177^Lu]Lu-PSMA-617 retained 22.3 ± 3.5% IA/g. The tumor-to-blood ratio reached high values as soon as 1 h p.i. and further increased to 619 and 2351 at 24 p.i. for [^177^Lu]Lu-P17 and [^177^Lu]Lu-P18, respectively, compared to 1306 for [^177^Lu]Lu-PSMA-617. Similar trends were also observed for the tumor-to-muscle, tumor-to-spleen, and tumor-to-kidney ratios (Table S1). The tumor-to-kidney ratios were below 1 for both [^177^Lu]Lu-P17 and [^177^Lu]Lu-P18, but improved to 9.8 for [^177^Lu]Lu-P17 at 24 h p.i., while the ratio remained under 1 for [^177^Lu]Lu-P18. For the PC3 flu tumors, retention was under 1.7% IA/g for both [^177^Lu]Lu-P17 and [^177^Lu]Lu-P18 at all time-points, clearly confirming their PSMA-specific uptake.

**Table 4:** Biodistribution^a^ in PC3-PIP and PC3-flu Tumor-Bearing Balb/c Nude Mice at 1, 2, and 24 h p.i. of [^177^Lu]Lu-P17

	1 h p.i.	2 h p.i.	24 h p.i.
blood	0.18 ± 0.10	0.09 ± 0.01	0.02 ± 0.02
heart	0.18 ± 0.02	0.07 ± 0.02	0.03 ± 0.01
lung	0.51 ± 0.06	0.53 ± 0.03	0.09 ± 0.06
**spleen**	**2.3** ± **0.4**	**0.81** ± **0.12**	**0.12** ± **0.01**
liver	0.15 ± 0.03	0.12 ± 0.03	0.25 ± 0.01
intestine	0.09 ± 0.01	0.06 ± 0.02	0.05 ± 0.03
**kidney**	**111** ± **33**	**50.3** ± **10.5**	**1.1** ± **0.1**
brain	0.02 ± 0.01	0.02 ± 0.01	0.01 ± 0.01
**tumorPC3-PIP**	**14.1** ± **0.6**	**14.1** ± **4.5**	**10.5** ± **0.3**
tumor PC3-flu	0.50 ± 0.38	0.17 ± 0.16	0.16 ± 0.02
muscle	0.10 ± 0.03	0.05 ± 0.10	0.01 ± 0.01
bone	0.16 ± 0.03	0.18 ± 0.01	0.79 ± 0.26
tail	0.66 ± 0.09	0.35 ± 0.05	0.28 ± 0.03

a Data are average ± SD % IA/g (*n* = 3).

**Table 5:** Biodistribution^a^ in PC3-PIP and PC3-flu Tumor-Bearing Balb/c Nude Mice at 1, 2, and 24 h p.i. of [^177^Lu]Lu-P18

	1 hp.i.	2 h p.i.	24 h p.i.
blood	0.19 ± 0.06	0.06 ± 0.01	0.01 ± 0.01
heart	0.36 ± 0.08	0.21 ± 0.02	0.03 ± 0.01
lung	1.3 ± 0.2	0.68 ± 0.12	0.13 ± 0.01
**spleen**	**14.6** ± **3.5**	**8.5** ± **1.0**	**0.89** ± **0.39**
liver	0.25 ± 0.01	0.16 ± 0.02	0.10 ± 0.02
intestine	0.20 ± 0.08	0.10 ± 0.01	0.02 ± 0.01
**kidney**	**147** ± **5**	**135** ± **11**	**25.4** ± **10.3**
brain	0.03 ± 0.01	0.02 ± 0.01	0.01 ± 0.01
**tumorPC3-PIP**	**19.8** ± **2.3**	**17.5** ± **2.3**	**17.7** ± **3.2**
tumor PC3-flu	0.62 ± 0.46	0.49 ± 0.51	1.6 ± 0.3
muscle	0.19 ± 0.05	0.14 ± 0.02	0.02 ± 0.01
bone	0.28 ± 0.01	0.63 ± 0.78	0.10 ± 0.08
tail	0.79 ± 0.08	0.77 ± 0.32	0.08 ± 0.01

a Data are average ± SD % IA/g (*n* = 3).

**Table 6:** Biodistribution^a^ in PC3-PIP and PC3-flu Tumor-Bearing Balb/c Nude Mice at 1, 2, and 24 h p.i. of [^177^Lu]Lu-PSMA-617

	1 h p.i.	2 h p.i.	24 h p.i.
blood	0.19 ± 0.01	0.03 ± 0.02	0.02 ± 0.03
heart	0.15 ± 0.09	0.02 ± 0.01	0.01 ± 0.01
lung	0.43 ± 0.19	0.08 ± 0.03	0.24 ± 0.23
**spleen**	**2.8** ± **2.2**	**0.38** ± **0.17**	**0.11** ± **0.01**
liver	0.12 ± 0.05	0.05 ± 0.02	0.12 ± 0.02
intestine	0.40 ± 0.34	0.04 ± 0.01	0.01 ± 0.01
**kidney**	**109±52**	**12.9±10.2**	**0.64** ± **0.18**
brain	0.03 ± 0.02	0.01 ± 0.01	0.01 ± 0.01
**tumorPC3-PIP**	**24.5** ± **13.0**	**20.4** ± **5.1**	**22.3** ± **3.5**
tumor PC3-flu	0.17 ± 0.13	0.05 ± 0.01	0.51 ± 0.18
muscle	0.15 ± 0.09	0.02 ± 0.01	0.01 ± 0.01
bone	0.29 ± 0.02	1.6 ± 1.5	0.04 ± 0.02
tail	0.75 ± 0.04	0.05 ± 0.10	0.03 ± 0.01

a Data are average ± SD % IA/g (*n* = 3).

As anticipated, both [^177^Lu]Lu-P17 and [^177^Lu]Lu-P18 were excreted renally, resulting in initially high tissue uptake in the kidneys (111 ± 33% for [^177^Lu]Lu-P17 and 147 ± 5% IA/g for [^177^Lu]Lu-P18 at 1 h p.i.). This uptake declined steeply over time, with values of 50.3 ± 10.5% for [^177^Lu]Lu-P17 and 135 ± 11% for [^177^Lu]Lu-P18 at 2 h p.i.; and further to 1.1 ± 0.1% for [^177^Lu]Lu-P17 and 25.4 ± 10.3% IA/g for [^177^Lu]Lu-P18 at 24 h p.i. Furthermore, the structural modifications of [^177^Lu]Lu-P18 resulted in a relatively high spleen uptake at 1 h p.i. (14.6 ± 3.5% IA/g) which was cleared by 24 h p.i. (0.89 ± 0.39% IA/g).

## Discussion

In this study, we evaluated the impact of 3-styryl-l-Ala in PSMA-targeting radioligand [^177^Lu]Lu-P17 and [^177^Lu]Lu-P18 in respect to their PSMA binding ability, affinity, and specificity. Both novel compounds were directly compared to commercially available [^177^Lu]Lu-PSMA-617 (Pluvicto) to assess their accumulation in PSMA-positive tumors as well as in nontargeted organs and tissues. The lower lipophilicity and molecular weight of the first linker building block, 3-styryl-l-Ala in P17, contrasted with the 2-naphthyl-l-Ala in PSMA-617. In P18, the second linker building block was modified by replacing the *trans*-cyclohexyl moiety with an aromatic 4-aminomethyl benzoic acid moiety. This aromatic moiety has been previously incorporated into other PSMA ligands, such as PSMA-624^5^ and PSMA-1007^7^, wherein the modification demonstrated significantly higher internalization rates in comparison to PSMA-617.^6^ However, the introduction of the 4-aminomethyl benzoic acid moiety also led to increased retention in nontargeted organs like the liver, gallbladder, and spleen. To mitigate these undesirable retentions, the 3-styryl-l-Ala moiety was employed.

The preparations of P17 and P18 were successfully carried out using a modified solid-phase synthesis distinct from the method used for PSMA-617. The main objectives of this modified synthesis were to (i) eliminate the use of hazardous triphosgene and (ii) improve the synthesis of the Glu-urea-Lys binding motif by reducing insoluble educts or components that typically hinder the solid-phase synthetic approach. The generation of 2-chlorotrityl chloride (2-CT) resin-immobilized and *tris*(*t*Bu)-protected Glu-urea-Lys (PSMA binding motif) followed four different strategies (Schemes S3, S7, and S8). 1. Original Method: this method converted H-Glu(O*t*Bu)-O*t*Bu to OCN-Glu(O*t*Bu)-O*t*Bu for conjugation with resin-immobilized lysine, allowing for the synthesis of approximately 300 mg of the PSMA binding motif. 2. Adjusted Method: this variation also converted H-Glu(O*t*Bu)-O*t*Bu to OCN-Glu(O*t*Bu)-O*t*Bu and enabled scaling up the PSMA binding motif to multigram scale (Scheme S7). 3. Alternative Method: this approach converted H-Glu(O*t*Bu)-O*t*Bu to CDI-adduct-Glu(O*t*Bu)-O*t*Bu, providing a nonhazardous strategy that avoids toxic triphosgene and offers a wider choice of solvents for synthesis (Scheme S8). 4. Preferred Strategy: this strategy applied for the synthesis of P17 and P18 involved the reversed conversion of H-Lys(Fmoc)-O*t*Bu to CDI-adduct-Lys(Fmoc)-O*t*Bu for further conjugation with resin-immobilized glutamate (Scheme S3). This final method also eliminates the need for triphosgene and enables a broader solvent selection. Additionally, it allows for the activation of both glutamate- and lysine-based building blocks if advantageous for the overall synthesis. Replacing the Alloc-protecting group in lysine with the Fmoc-protecting group is preferred, as Alloc-deprotection is more complicated, time-consuming, and expensive.

The conjugation of Fmoc-protected *trans*-4-(Fmoc-aminomethyl)cyclohexanecarboxylic acid (Fmoc-TXA) in P17 was also improved compared to the original synthesis of PSMA-617 that used the same building block. The original method employed HBTU, DMF, and DIPEA for the conjugation of Fmoc-TXA,^5,6^ while the alternative method utilized DIC, Pure, DMF, and DCM (Schemes S4 and S5). This modification aimed to eliminate the undesirable use of HBTU, which can partially convert Fmoc-TXA into an insoluble gel-like solid upon activation, reducing the overall yield of the reaction. As a result, the conjugation of Fmoc-TXA often needed to be performed twice, leading to longer preparation times and increased costs.

The conjugation of the DOTA moiety was successfully achieved using an alternative synthesis. The original method utilized DOTA-*tris*(*t*Bu)ester, HBTU, DMF, and DIPEA,^5,6^ while the alternative method employed active DOTA-*tris*(*t*Bu)-NHS ester, DMF, and TEA (Scheme S6). Both conjugation routes are standard and are typically chosen based on the overall synthetic strategy, including factors like the orthogonality of protecting groups and the stability of the solid phase. Notably, the alternative strategy offers a significant advantage by avoiding HBTU, which can “cap” unreacted free amines. This improved synthetic approach can be effectively utilized by both academic institutions and industrial organizations in the synthesis of various established and novel PSMA ligands.

The amount of TFA associated with both compounds was determined by NMR to mitigate errors in concentration-dependent assays. With a molecular weight of TFA (114.0 g/mol), the association of 2.4 equiv of TFA with P17 and P18 (1018.2 and 1012.2 g/mol, respectively) increases their molecular weights by over 25%. This factor is often overlooked, despite its substantial impact on both in vitro and in vivo data.

For the transfected cell line PC-3 PIP, the IC_50_ values were found to be10-fold higher than those for native LNCaP and LNCaP-derived C4-2 cell lines. This difference is presumably due to PC3 being a filamin A-positive cell line. Anilkumar et al. demonstrated that filamin A associates with the cytoplasmic tail of PSMA, affecting its localization to the recycling endosomal compartment. This phenomenon significantly influences the binding and internalization of PSMA and its *N*-acetylated, α-linked acidic dipeptidase activity.^15^ Thus, despite PC3-PIP having approximately 10-fold higher PSMA expression as described by Banerjee et al.,^16^ the binding affinity remains reduced.

Interestingly, the crystal structures of PSMA–ligand complexes revealed differential binding modes for the modified linkers and DOTA chelator functionalities within the PSMA binding pocket. This provides a foundation for the future rational design of PSMA ligands. However, it is important to note that the inhibitors used for determining the structures of the PSMA–inhibitor complexes featured metal-free DOTA chelators. The structures of DOTA chelators complexed with a metal ion may differ from the metal-free DOTA structures reported here, and future studies are anticipated to shed light on these issues. Consequently, it can be hypothesized that interactions between the DOTA chelators and PSMA, particularly with the W541 side chain as observed in the PSMA-P17 and PSMA-P18 complexes, may also vary. An altered interaction pattern between PSMA and a metal-loaded inhibitor could influence both the inhibitor’s potency and its biological characteristics.

Overall, the binding affinities, internalizations, and tumor uptakes of [^177^Lu]Lu-P17 and [^177^Lu]Lu-P18 were found to be comparable to those of [^177^Lu]Lu-PSMA-617. However, a striking difference was observed in the accumulation of [^177^Lu]Lu-P18 in the spleen, which is likely associated with the changes in aromaticity and lipophilicity^7,17^ (∼2–3% IA/g for [^177^Lu]Lu-PSMA-617 and [^177^Lu]Lu-P17 in comparison to ∼14% IA/g for [^177^Lu]Lu-P18 at 1 h p.i.). Another crucial organ are the kidneys, where the clearance rates for [^177^Lu]Lu-PSMA-617 and [^177^Lu]Lu-P17 were significantly faster than for [^177^Lu]Lu-P18. Specifically, the uptake decreased from ∼110% IA/g at 1 h p.i. to ∼1% IA/g at 24 h p.i. for both [^177^Lu]Lu-PSMA-617 and [^177^Lu]Lu-P17, whereas [^177^Lu]Lu-P18 decreased from ∼150% IA/g at 1 h p.i. to ∼25% IA/g at 24 h p.i. At this point, it is important to note that human and mouse PSMA are not structurally identical, which may influence how PSMA-targeting ligands interact with the human versus mouse protein expressed in tumor versus nontarget tissues and organs.^18^ This variability can also influence the uptake, retention, and clearance in nontarget tissues and organs, such as the kidneys, to some extent.

Based on the aforementioned data, [^177^Lu]Lu-P17 emerges as a promising candidate, exhibiting favorable tumor uptake and tumor-to-background ratios. The lower lipophilicity of P17 could be strategically tailored for various applications while maintaining advantageous pharmacokinetic and biodistribution properties. This approach may be particularly beneficial when employing a more lipophilic chelator than DOTA. A prominent example could be the use of the Macropa chelator with two aromatic picolinates for the coordination of the highly potent alpha-emitter Ac-225.^19^ Not only this approach, but also many others confirm the key role of the linker in the development of highly potent PSMA ligands. For instance, Lundmark et al.^20^ investigated the derivatization of the PSMA-617 linker. The resulting [^111^In]In-BQ7859, with a 2-naphthyl-l-alanine and l-tyrosine linker, exhibited favorable targeting and improved the tumor-to-blood ratio. Kazuta et al.^21^ developed PSMA-targeting radioligand incorporating dual-functional linkers: a hydrophilic linker, d-glutamic acid, and a hydrophobic linker, AMBA, which enhanced pharmacokinetics. Similarly, Uspenskaya et al.^22^ focused on a phenylalanine/tyrosine-based dipeptide linker with mixed chiral centers and substituted aromatic fragments, revealing that the optimal linker contained l-phenylalanine at the *N*-terminus of the dipeptide chain. In another approach, Huang et al.^23^ aimed to reduce the accumulation of PSMA ligands in salivary glands, demonstrating that [^68^Ga]Ga-JB-1498, with a highly negatively charged linker, significantly decreased salivary gland uptake. Finally, a broader strategy to improve the therapeutic index through cleavable linkers is discussed in the review by Lau et al.^24^ In summary, next-generation theranostic agents should exhibit high receptor specificity, strong affinity (i.e., high binding potential), stereoselectivity, rapid internalization, good tumor penetration and distribution, minimal host immune response, and low to moderate lipophilicity to ensure favorable pharmacokinetics. Additionally, they should circulate quickly through the body, clear rapidly from background organs and tissues, be easy to produce, allow straightforward radiolabeling, and demonstrate high radiolytic and enzymatic stability.

## Conclusions

In this study, we evaluated the biological properties of [^177^Lu]Lu-PSMA-617 alongside its novel analogs, [^177^Lu]Lu-P17 and [^177^Lu]Lu-P18. These analogs feature modified lipophilic linkers, specifically substituting the 2-naphthyl-l-Ala moiety of PSMA-617 with a less lipophilic 3-styryl-l-Ala. The compounds were prepared using an optimized solid-phase synthesis method. Despite these linker modifications, no significant differences were observed in PSMA inhibition potency, cellular binding affinity, internalization rates, or in vivo biodistribution studies indicating comparable tumor uptake across all three compounds. The first crystal structure of the PSMA/PSMA-617 complex was reported. Notably, crystal structures of the PSMA–ligand complexes unveiled distinct binding modes for the modified linkers and DOTA chelator functionalities within the PSMA binding pocket. These insights underscore the potential of linker modifications to enhance the pharmacokinetic properties of PSMA ligands, facilitating the development of innovative theranostic agents for PCa.

## Experimental Section

### Chemicals and Radionuclides

All chemicals (>95% pure, ultrapure for radiolabeling) and solvents (HPLC grade, metal-free for radiolabeling) were purchased from abcr, Bachem, Carbolution, CheMatech, Fluka, Iris Biotech, Macrocyclics, Merck Group, Carl Roth, or Sigma-Aldrich and were used without further purification.

No-carrier-added ^177^LuCl_3_ in 0.04 M HCl was purchased from Isotope Technologies Munich (ITM), München, Germany.

### Solid-Phase Synthesis

PSMA-617 was synthesized using 2-CT resin support as previously reported.^6^ For P17 and P18, four different synthetic strategies for synthesizing the PSMA binding motif (i.e., resin-immobilized and *tris*(*t*Bu)-protected Glu–urea–Lys) on a solid phase were employed and compared. The ensuing coupling of the linker was performed according to the standard fluorenylmethoxycarbonyl (Fmoc) protocol. Finally, conjugation of the DOTA chelator was realized using HBTU-activated DOTA-*tris*(*t*Bu)ester or an activated DOTA-NHS ester.

### Purification and Quality Control

P17 and P18 were purified by semipreparative HPLC and analyzed by analytical HPLC, ESI-MS, and MALDI-MS as well as by 1D ^1^H, ^13^C, and ^15^N NMR and 2D NMR techniques, viz. COSY, HSQC, and HMBC.

For semipreparative and analytical HPLC, a LATEK P-402 pump coupled to a Merck Hitachi L-7420 UV/vis signal detector was employed. Column: NUCLEODUR HILIC (Macherey Nagel), 5 μm, 110 Å, 250 × 21 mm; Eluents: (A) 97% ACN + 0.2% formic acid (FA), (B) water + 0.2% FA; solvent gradient: 0–40 min 3–50% B; flow: 15 mL/min; wavelength: 214 nm; Temperature: RT.

MS of P17 and P18 were acquired using an Esquire 600 (Bruker) MS instrument equipped with an ion trap and an LT2 Plus (Scientific Analysis Instruments, SAI) MS instrument equipped with a time-of-flight (TOF) detector.

### NMR

NMR spectra of P17 (9.9 mg) and P18 (11.7 mg) in DMSO-*d*_6_ at 25 °C were acquired using Bruker Avance Neo and Avance III instruments equipped with 5 mm, inverse-configuration, helium-cooled and 5 mm, normal-configuration (cryo)probes, respectively, both with *z*-axis gradient capability at field strengths of 16.45 and 9.4 T, respectively, operating at 700.13 and 176.05 MHz for ^1^H and ^13^C nuclei, respectively, and 400.26, 376.62, 100.64, and 40.56 MHz for ^1^H, ^19^F, ^13^C, and ^15^N nuclei, respectively. Pulse widths were calibrated following the described protocol.^25^ General NMR experimental and acquisition details for 1D ^1^H, ^19^F, ^13^C, ^13^C-DEPT, ^15^N-INEPT, and ^1^H{^1^H}-homodecoupling and standard, gradient-selected 2D ^1^H{^1^H}-COSY, ^1^H{^13^C/^15^N}-HSQC, and ^1^H{^13^C}-HMBC have been previously described for routine spectral assignment and structural analysis.^26^ 2D spectra were generally acquired using NUS. The chemical shifts of ^1^H and ^13^C nuclei are referenced relative to the solvent signals (δ_1H,DMSO-*d*_5__ = 2.50 ppm and δ_13C,DMSO-*d*_6__ = 39.52 ppm) while the chemical shifts of ^15^N nuclei are referenced relative to external 90% CH_3_NO_2_ in CD_3_NO_2_ (δ_15N_ = 0 ppm).

### Determination of the Extent of Protonation

The extent of protonation of the analytes was based on the number of TFA ions per analyte molecule. First, the relative NMR solution concentrations for each analyte was determined in comparison to a reference sample of 1,1,1-trifluoroethanol (TFE) in DMSO-*d*_6_ by ^1^H NMR using identical acquisition parameters and integrating a signal of one proton in the spectrum of the analyte relative to the integration of the methylene proton signal of TFE. Likewise, the relative NMR solution concentrations of TFA ions for each analyte were determined in comparison to the same reference sample of TFE by ^19^F NMR using identical acquisition parameters and integrating the signal of the TFA fluorine signal of the analyte sample relative to the integration of the methyl fluorine signal of TFE. The ratio of the two relative concentrations then yields the number of TFA ions per analyte molecule.

### Expression and Purification of Recombinant Human PSMA

Expression and purification of the extracellular part of human PSMA (amino acids 44–750) were performed essentially as described previously.^27^ Briefly, the N-terminally tagged PSMA fusion was overexpressed in Schneider’s S2 cells and the conditioned medium concentrated and dialyzed by tangential flow filtration (Millipore, Mosheim, France). The recombinant protein was purified by the combination of affinity chromatography on Streptactin Sepharose (IBA, Göttingen, Germany) and size-exclusion chromatography on a Superdex 200 column (GE Healthcare Bio-Sciences, Uppsala, Sweden) with the mobile phase composed of 20 mM Tris.HCl and 150 mM NaCl (pH 8.0).

### Crystallization and Data Collection

Diffraction-quality crystals were grown using the hanging-drop vapor diffusion method at 293 K. Purified PSMA (10 mg/mL) was mixed with a given inhibitor dissolved in 50 mM Tris·HCl and 150 mM NaCl (pH 8.0) at a 1:19 ratio. The protein–inhibitor solution was then mixed with an equal volume of mother liquor [33–34% (v/v) pentaerythritol propoxylate PO/OH 5/4 (Sigma-Aldrich), 1% (w/v) PEG 3350, and 100 mM Tris.HCl (pH 8.0)] and 1 μL crystallization droplets were equilibrated against 750 μL of the precipitant solution in pregreased Crystalgen SuperClear plates. Rectangular crystals were vitrified directly from the droplets in liquid nitrogen. Diffraction data were collected from a single crystal using synchrotron radiation at the MX14.1 and MX14.2 beamlines (0.91841 Å; BESSYII, Berlin, Germany) equipped with a Pilatus 6 M detector (Dectris, Switzerland) at 100 K. Data sets were indexed, integrated, and scaled using the XDSAPP interface.^28^

### Structure Refinement

Difference Fourier methods were used to determine the structures of the PSMA–inhibitor complexes with ligand-free GCPII (PDB code 2OOT) used as a starting model.^29^ Iterative refinement and model building cycles were performed using Refmac 5.8.0135^30^ and Coot graphics package,^31^ respectively. Ligand topologies and coordinates were generated with Acedrg^32^ and inhibitors were fitted into the |Fo| – |Fc| electron density maps in the final stages of the refinement. Approximately 1000 randomly selected reflections were retained for cross-validation (*R*_free_) during the refinement process. The final models were validated using the wwPDB X-ray validation server^33^ and deposited in the Protein Data Bank (PDB) under accession codes 8BOW, 8BO8, and 8BOL for PSMA-617, P17, and P18, respectively. Data collection and structure refinement statistics are summarized in Tables 7 and 8.

**Table 7:** Data Collection Statistics^a^

collection statistics			
inhibitor	617	P17	P18
PDB code	8BOW	8BO8	8BOL
wavelength (Å)	0.918	0.918	0.918
space group	*I*222	*I*222	*I*222
unit-cell parameters *a*, *b*, *c* (Å)	101.28, 130.00, 159.492	101.89, 130.29, 159.70	101.69, 130.25, 159.501
resolution limits (Å)	50.00–1.58 (1.67–1.58)	50.01–1.55 (1.64–1.55)	50.01–1.55 (1.64–1.55)
number of unique reflections	143,725 (22,658)	152,978 (24,349)	152,652 (24,271)
redundancy	5.2 (5.0)	6.6 (6.2)	6.6 (6.2)
completeness (%)	99.5 (97.9)	99.8 (98.9)	99.8 (98.9)
*I*/σ*I*	17.86 (1.98)	10.87 (0.54)	12.82 (0.88)
*R*_merge_	0.055 (0.71)	0.102 (2.77)	0.082 (1.663)
CC1/2	0.999 (0.805)	0.999 (0.449)	0.999 (0.664)

a Values in parentheses are for the highest resolution shells.

**Table 8:** Data Refinement Statistics^a^

refinement statistics			
resolution limits (Å)	45.25–1.58 (1.62–1.58)	50.01–1.55 (1.59–1.55)	50.01–1.55 (1.59–1.55)
total number of reflections	141,700 (10,179)	145,207 (11,105)	144,982 (10,999)
number of reflections in working set	139,600 (10,030)	137,488 (10,567)	137,357 (10,453)
number of reflections in test set	2100 (149)	7719 (538)	7625 (546)
*R*/*R*_free_ (%)	18.05/19.79 (33.1/32.9)	20.16/22.63 (42.1/43.8)	19.19/21.35 (37.4/37.8)
total number of non-H atoms	6787	6614	6644
number of non-H protein atoms	5830	5767	5775
number inhibitor molecules	1	1	1
number of water molecules	635	526	548
**average B-factor(Å**^**2**^**)**	26.28	32.22	30.42
protein atoms	23.89	30.33	28.47
waters	34.90	37.856	36.78
inhibitor	50.92	31.70	34.41
b**Ramachandran plot (%)**			
most favored	98	98	98
additionally allowed	2	2	2
disallowed	val 382	val 382, Gly 335	val 382
**R.m.s. deviations:**			
bond lengths (Å)	0.011	0.015	0.014
bond angles (deg)	1.51	1.68	1.62
planarity (Å)	0.008	0.008	0.008
chiral centers (Å^3^)	0.097	0.111	0.112
missing residues	AA 44–55, AA 654–656	AA 44–55, AA 654–655	AA 44–55, AA 654–655

a Values in parentheses are for the highest resolution shells.

b Structures were analyzed using the wwPDB X-ray validation server.^33^

### Radiolabeling and Quality Control

^177^LuCl_3_ was reformulated in sodium acetate (0.4 M, pH 4.5) to attain a concentration of ∼2 MBq/μL (∼54.1 μCi/μL). Each PSMA ligand, 1 mM stock solution in DMSO/water at 1:9 (v/v), was added to the ^177^LuCl_3_ solution to attain a molar activity of 1–10 MBq/nmol and the reaction mixture incubated for 30 min at 95 °C. Quality control was performed by reversed-phase thin-layer chromatography (RP-TLC) and radio-RP-HPLC.

### Radio-Analytical RP-HPLC and RP-TLC

[^177^Lu]Lu-P17 and [^177^Lu]Lu-P18 were analyzed by a Thermo Fisher Ultimate 3000 HPLC equipped with a variable wavelength detector and an Elysia-Raytest Gabi flow cell gamma detector. Column: Chromolith C18 end-capped, 2 μm, 130 Å, 100 × 4.6 mm; Eluents: (A) water + 0.1% TFA, (B) ACN + 0.1% TFA; Solvent gradient: 0–15 min 95–5% A; Flow 2 mL/min; Wavelength: 254 nm; Temperature: RT. In addition, both compounds were analyzed over silica gel 60 RP-18 plates (Merck, Darmstadt, Germany) using 0.1 M sodium citrate (pH 8) as the mobile phase.

### *n*-Octanol/PBS Distribution Coefficients

The lipophilicities of [^177^Lu]Lu-PSMA-617, [^177^Lu]Lu-P17 and [^177^Lu]Lu-P18 were determined by their equilibrium distribution in a two-phase system consisting of n-octanol and PBS (pH 7.4). Small aliquots from both phases were collected and analyzed in a gamma counter (Cobra Autogamma B5003, Canberra, Packard; Frankfurt, Germany) in order to calculate distribution coefficients log *D*. The experiment was repeated in three independent assays performed in triplicate.

### Cell Lines

Cell culture media and supplements were purchased from PAN Biotech if not stated otherwise. In vitro assays were performed using the sublines of the androgen-independent PC3 human PCa cell line, PSMA-positive (PSMA+) PC3-PIP and PSMA-negative (PSMA−) PC3-flu cells, purchased from Johns Hopkins University (Baltimore, MA, USA). Both cell lines were housed in a humidified atmosphere of 5% CO_2_ at 37 °C and cultured in RPMI-1640 medium supplemented with 10% fetal calf serum (FCS), 1% l-glutamine (l-Gln), and puromycin (2 μg/mL). In addition, cell-binding affinity assays were also performed on PSMA + LNCaP (ATCC CRL-1740) and C4-2 (ATCC CRL-3314) cell lines. LNCaP was derived from a human lymph node metastatic lesion of PCa while C4-2 was isolated from an LNCaP-based tumor xenograft of an androgen-depleted mouse. Both cell lines were cultured as described above but in the absence of puromycin. Routine cell culture was performed twice a week at RT using phosphate-buffered saline (PBS, pH 7.4) for washing and 0.05% trypsin for cell detachment.

### Animals

For in vivo experiments, 5–7 week-old male athymic BALB/c nu/nu mice weighing 18–20 g were obtained from Charles River Laboratories (Sulzfeld, Germany). All mice were housed in groups under standard conditions in approved facilities with 12 h light/dark cycles and given food and water ad libitum. After at least 1 week of acclimatization and when reaching the age of 8 weeks, mice were subcutaneously inoculated into the right trunk with 6 × 10^6^ PC3-PIP cells in 100 μL HBSS and into the left trunk with 5 × 10^6^ PC3-flu cells in 100 μL HBSS. By injecting both cell lines in one animal, interindividual variation in the specific uptake of the respective radioligand could be minimized and, importantly, the number of animals used was reduced. All animals were monitored for tumor growth, weight, and their general health status at least twice a week. Biodistribution studies (*n* = 3 per radioligand and time point) were performed when the tumor xenografts reached 7–11 mm in diameter and were comparable in size. All animal experiments were approved by the responsible local authorities (Regierungspräsidium Karlsruhe, Germany; permit number: 35-9185.81/G-264/21) in compliance with the current laws of the Federal Republic of Germany.

### Inhibitor Potency In Vitro

The inhibitory potency was determined using a fluorescence-based assay described previously.^11^ Briefly, 20 pM recombinant human PSMA was preincubated with a 3-fold dilution series of inhibitors in 50 mM Tris.HCl, 150 mM NaCl, and 0.001% C_12_ E_8_ for 10 min at 37 °C. The reaction was initiated by the addition of 100 nM substrate in a total volume 50 μL. Following 15 min incubation at 37 °C, the reaction was stopped by the addition of 50 μL of 0.1% TFA in 5% ACN. Reaction mixtures were analyzed by RP-HPLC (Shimadzu, HPLC Prominence system) equipped with a Kinetex 2.6 μm XB-C18 100 Å column and a fluorescence detector (λ_EX_/λ_EM_ = 492/516). HPLC separation was performed using a linear solvent gradient over 11 min from 20 to 50% of eluent B (0.1% (v/v) TFA in ACN) at a flow rate of 0.6 mL/min. The mobile phase A was 0.1% (v/v) TFA in water. The noninhibited reaction was assigned 100% and the reaction without enzyme present was defined as 0%. Data were fitted using a nonlinear regression algorithm (GraphPad Prism Software, version 8) to calculate IC_50_ values from the inhibition curves. Three independent assays were performed for each compound.

### Cell-Binding Affinity Assay

The binding affinities of PSMA-617, P17, and P18 were determined using a competitive assay with [^177^Lu]Lu-PSMA-617. All three compounds were incubated at 12 different concentrations (0, 0.5, 1, 2.5, 5, 10, 25, 50, 100, 500, 1,000, and 5000 nM) with 0.75 nM [^177^Lu]Lu-PSMA-617 on either PSMA + cell lines PC3-PIP, LNCaP, or C4-2 or the PSMA– cell line PC3-flu plated at a concentration of 10^6^ cells/well. After 45 min incubation at RT, the radioligand was removed and cells washed twice with 100 μL and once with 200 μL ice-cold PBS. The activity specifically accumulated in the cells was measured in a gamma counter (Cobra Autogamma B5003, Canberra, Australia; Packard, Frankfurt, Germany). Data were fitted using a nonlinear regression algorithm (GraphPad Prism Software, version 8) as described above. The experiment was repeated in three independent assays performed in triplicate.

### Cellular Internalization Assay

The internalization rates of [^177^Lu]Lu-PSMA-617, [^177^Lu]Lu-P17, and [^177^Lu]Lu-P18 were measured using either PC3-PIP or PC3-flu cells. Cells (10^5^ cells/well) were seeded in poly-l-lysine (PLL)-coated plates 24 h before the experiment. The cells were incubated for 45 min at 37 °C with 30 nM [^177^Lu]Lu-P17, [^177^Lu]Lu-P18, or [^177^Lu]Lu-PSMA-617 in 250 μL Opti-MEM (Gibco). Another set of cells was treated analogically while the PSMA-inhibitor 2-(phosphonomethyl)pentane-1,5-dioic acid (2-PMPA; 100 mM, 500 μM/well) was added in order to subtract the nonspecific binding. The respective radioligand was removed and cells were washed three times with 1 mL ice-cold PBS and the surface-bound radioactivity detached by incubating the cells twice with 500 μL glycine buffer (50 mM, pH 2.8) for 5 min. After washing the cells once with 1 mL ice-cold PBS, the internalized fraction was determined by subsequent cells lysis with 500 μL NaOH (0.3 M, pH 14). The collected glycine and hydroxide fractions were measured in a gamma counter (Cobra Autogamma B5003, Canberra, Australia; Packard, Frankfurt, Germany) and calculated as % AA specifically bound on either the cells’ surfaces or internalized inside the cells. Three independent assays were performed.

### Biodistribution Studies

Anaesthetized PC3-PIP- and PC3-flu-tumor-bearing mice (1.5% isoflurane, Abbott) were intravenously injected with either [^177^Lu]Lu-PSMA-617, [^177^Lu]Lu-P17, or [^177^Lu]Lu-P18 (0.8–1.8 MBq, 0.6–1.0 nmol, 100 μL). The mice were sacrificed 1, 2, or 24 h pi. Groups of three mice were used for each time-point. Selected tissues and organs were collected, weighed, and measured in a gamma counter (Cobra Autogamma B5003, Canberra, Australia; Packard, Frankfurt, Germany). The results were calculated as % IA/g.

## Supporting Information Available

Molecular Formula Strings, general schemes for the solid-page synthesis of P17 (Scheme S1) and P18 (Scheme S2), synthesis of the binding motif for both P17 and P18 (Scheme S3), linker coupling for P17 (Scheme S4) and P18 (Scheme S5), chelator conjugation for both P17 and P18 (Scheme S6), quality control with MS (Figures S1–S6), quality control with HPLC (Figures S7–S9), NMR data and description (Figure S10–S21), quality control with radio-HPLC (Figures S22–S24), biodistribution data (Figures S25 and S26), tumor-to-organ ratios (Table S1), adjusted methods for the preparation of resin-immobilized Glu-urea-Lys for P17 and P18 (Schemes S7 and S8) (PDF)

## Abbreviations used

1D: one-dimensional
2CT: 2-chlorotrityl chloride resin
2D: two-dimensional
2-PMPA: 2-(phosphonomethyl)pentane-1,5-dioic acid
ACN: acetonitrile
Ala: alanine
Alloc: *N*-allyloxycarbonyl
AMBA: 4-(Fmoc-aminomethyl)-benzoic acid
Arg: arginine; Asn, asparagine
CDI: *N*,*N*′-carbonyldiimidazole
COSY: correlation spectroscopy
DCM: dichloromethane
DEPT: distortionless enhancement by polarization transfer
DIC: *N*,*N*′-diisopropylcarbodiimide
DIPEA: *N*,*N*-diisopropylethylamine
DMF: dimethylformamide
DMSO: dimethyl sulfoxide
DOTA: 1,4,7,10-tetraazacyclododecane-1,4,7,10-tetraacetic acid
EMA: European Medicines Agency
ESI-MS: electrospray ionization-mass spectrometry
FA: formic acid
FCS: fetal calf serum
FDA: U.S. Food and Drug Administration
Fmoc: 9-fluorenylmethylmethoxycarbonyl
Gln: glutamine
Glu: glutamate
Gly: glycine
HBTU: 2-(1*H*-benzotriazol-1-yl)-1,1,3,3-tetramethyluronium hexafluorophosphate
HMBC: heteronuclear multiple bond correlation
HPLC: high-performance liquid chromatography
HSQC: heteronuclear single quantum correlation
IC_50_: half-maximum inhibitory concentration
INEPT: insensitive nuclei enhanced polarization transfer
i.v: intravenously
log *D*: logarithm of distribution coefficient
Lys: lysine; *m*/*z*, mass-to-charge ratio
MALDI-MS: matrix-assisted laser desorption ionization-mass spectrometry
mCRPC: metastatic castration-resistant prostate cancer
NMR: nuclear magnetic resonance
PBS: phosphate-buffered saline
Phe: phenylalanine
p.i: postinjection
PCa: prostate cancer
PLL: poly l-lysine
PSMA: prostate-specific membrane antigen
r.m.s: root mean squared
RP: reversed-phase
RT: room temperature
SAR: structure–activity relationship
SD: standard deviation
*t*Bu: *tert*-butyl
TFA: trifluoroacetic acid
TFE: trifluoroethanol
TOF: time-of-flight
Thr: threonine
TLC: thin layer chromatography
Trp: tryptophan
TXA: *trans*-4-(aminomethyl)cyclohexanecarboxylic acid
Å: Ångström
μ: micro; σ, sigma
°C: degrees Celsius
% AA: percentage of total applied activity
% IA/g: percentage of the injected activity per gram of tissue mass.
