# Supplementary material for: Structure–Activity Relationships and Biological Insights into PSMA-617 and Its Derivatives with Modified Lipophilic Linker Regions
Source: ACS Omega. 2025 Feb 12;10(7):7077–90. doi: 10.1021/acsomega.4c10142 (PMC11865982; doi:10.1021/acsomega.4c10142)
Supplement: Supplementary file 1 — ao4c10142_si_001.pdf [file ao4c10142_si_001.pdf]

Structure–Activity Relationships and Biological Insights into PSMA-617 and Its Derivatives with Modified Lipophilic Linker Regions

Martin Schäfer[1], Ulrike Bauder-Wüst[2], Mareike Roscher[1], Lucia Motlová[3], Zsófia Kutilová[3], Yvonne Remde[1], Karel D. Klika[4], Jürgen Graf[5], Cyril Bařinka[3], and Martina Benešová-Schäfer[2*]

1. Service Unit for Radiopharmaceuticals and Preclinical Studies, German Cancer Research Center (DKFZ), Im Neuenheimer Feld 280, 69120 Heidelberg, Germany

2. Research Group Translational Radiotheranostics, German Cancer Research Center (DKFZ), Im Neuenheimer Feld 280, 69120 Heidelberg, Germany

3. Laboratory of Structural Biology, Institute of Biotechnology of the Czech Academy of Sciences, Průmyslová 595, 25250 Vestec, Czech Republic

4. Molecular Structure Analysis, German Cancer Research Center (DKFZ), Im Neuenheimer Feld 280, 69120 Heidelberg, Germany

5. Nuclear Magnetic Resonance Laboratory, Institute of Organic Chemistry, Heidelberg University, Im Neuenheimer Feld 270, 69120 Heidelberg, Germany

E-Mail addresses:

martin.schaefer@dkfz-heidelberg.de; u.bauder-wuest@dkfz-heidelberg.de;

mareike.roscher@dkfz-heidelberg.de; lucia.motlova@ibt.cas.cz; zsofia.kutil@ibt.cas.cz;

y.remde@dkfz-heidelberg.de; klikakd@yahoo.co.uk; graf@oci.uni-heidelberg.de;

Cyril.Barinka@ibt.cas.cz; m.benesova@dkfz-heidelberg.de.

Classification: Restricted

***Correspondence to**:

Dr. Martina Benešová-Schäfer

German Cancer Research Center (DKFZ)

Foundation under Public Law

Im Neuenheimer Feld 280

69120 Heidelberg

Germany

E-mail: m.benesova@dkfz-heidelberg.de

Phone: +49-6221-42-5355

Fax: +49-6221-42-5356

Classification: Restricted

Molecular Formula Strings

	SMILES	Molecular weight [g/mol]	IC_{50} [nM]
**P17**	O=C(CC[C@@H](C(O)=O)NC(N[C@@H](CCCCNC([C@H](C/C=C/C1=CC=CC=C1)NC([C@@H]2CC[C@@H](CNC(CN3CCN(CC(O)=O)CCN(CC(O)=O)CCN(CC(O)=O)CC3)=O)CC2)=O)=O)C(O)=O)=O)O	1,018.1	0.30 ± 0.04
**P18**	O=C(CC[C@@H](C(O)=O)NC(N[C@@H](CCCCNC([C@H](C/C=C/C1=CC=CC=C1)NC(C2=CC=C(CNC(CN3CCN(CC(O)=O)CCN(CC(O)=O)CCN(CC(O)=O)CC3)=O)C=C2)=O)=O)C(O)=O)=O)O	1,012.1	0.45 ± 0.09
**PSMA-617**	O=C(CC[C@@H](C(O)=O)NC(N[C@@H](CCCCNC([C@H](CC(C=C1)=CC2=C1C=CC=C2)NC([C@@H]3CC[C@@H](CNC(CN4CCN(CC(O)=O)CCN(CC(O)=O)CCN(CC(O)=O)CC4)=O)CC3)=O)=O)C(O)=O)=O)O	1,042.2	0.05 ± 0.03

Classification: Restricted

Scheme S1: General scheme for the solid-phase synthesis of P17.

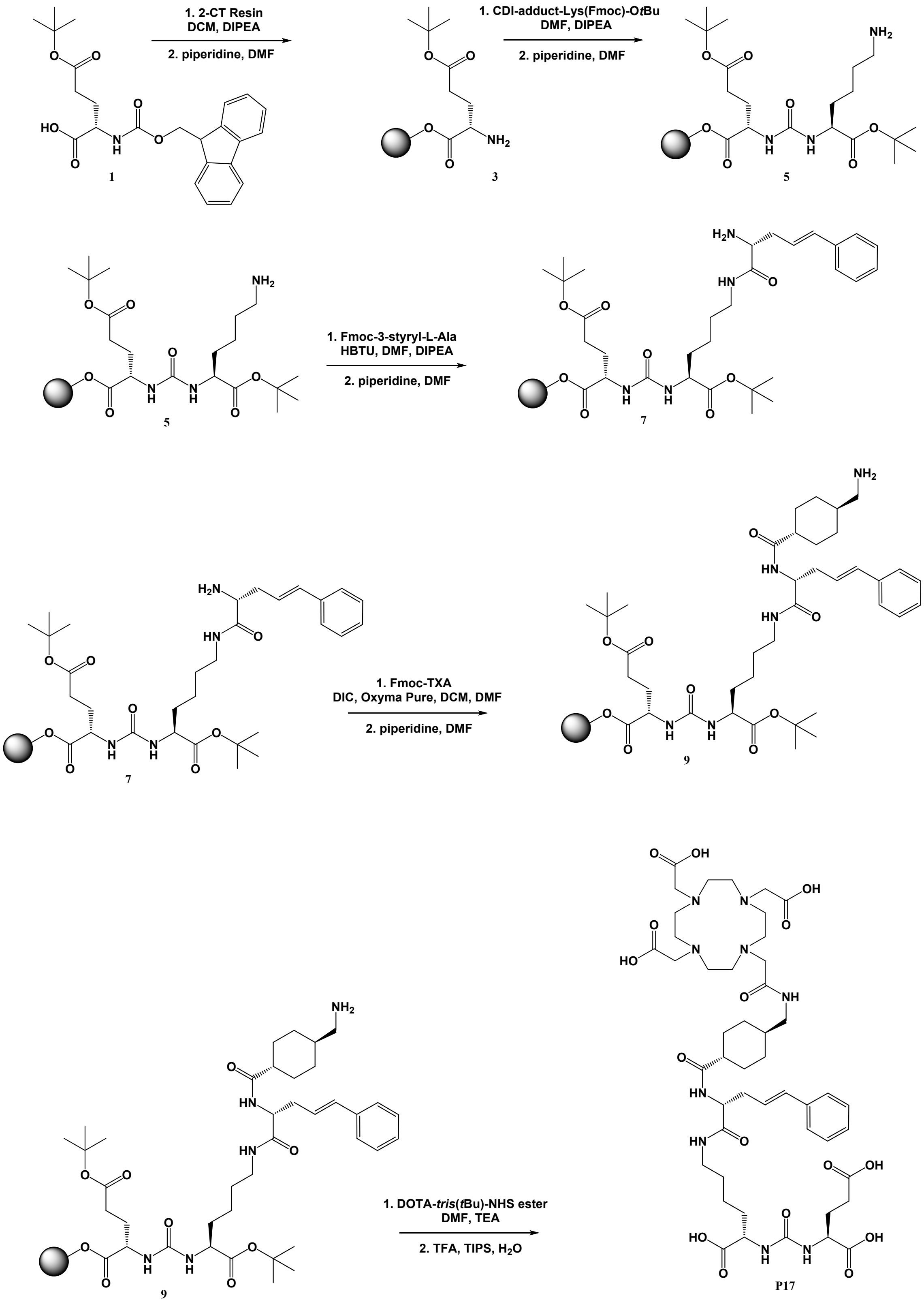

Classification: Restricted

Scheme S2: General scheme for the solid-phase synthesis of P18.

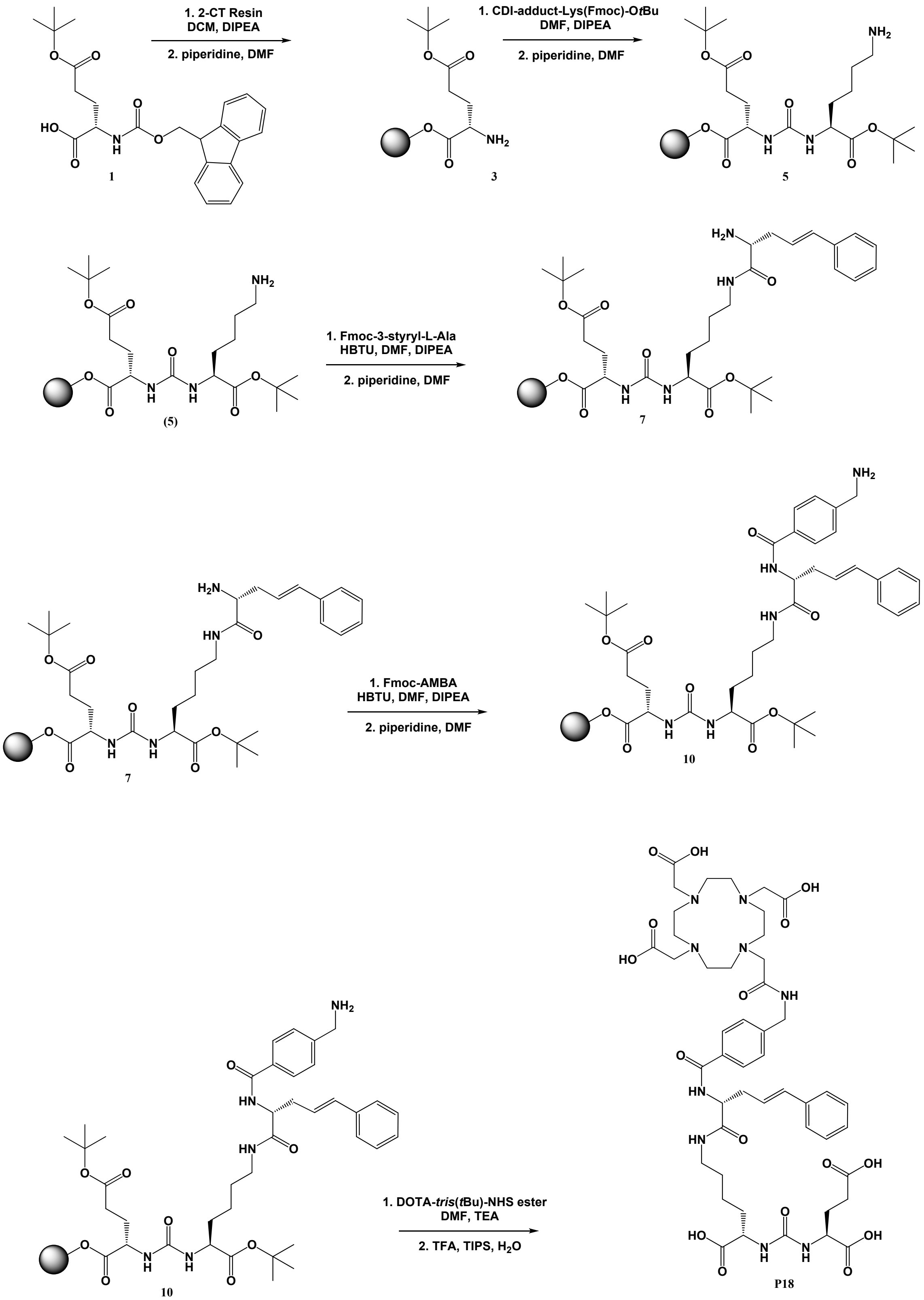

Classification: Restricted

Synthesis of the binding motif for both P17 and P18

Scheme S3: Synthesis of the peptidomimetic glutamate-urea-lysine via adapted methodology.

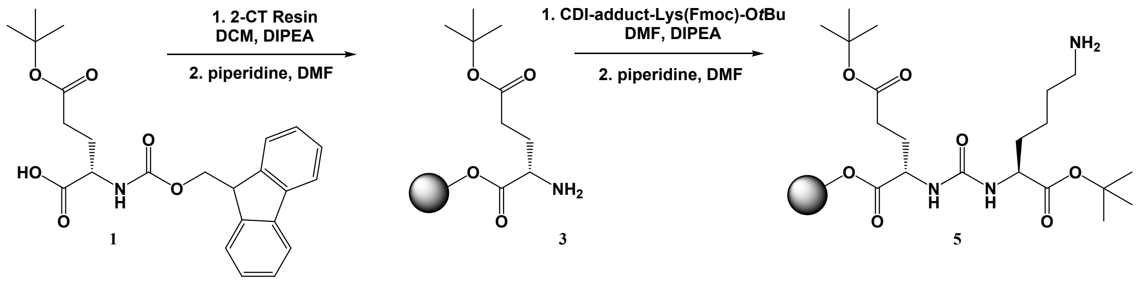

2-CT resin (5 g, 6.1 mmol, substitution capacity 1.22 mmol/g, 100–200 mesh) was first agitated in dry DCM for 45 min and then washed with dry DCM followed by reaction with 1.2 equiv Fmoc-Glu(O*t*Bu)-OH **1** and 4.8 equiv DIPEA in 150 mL of dry DCM. The coupling of the amino acid onto the resin proceeded with gentle agitation over the course of 16 h.

The glutamate-immobilized resin **2** was then washed with DCM with unreacted chlorotrityl groups in the resin blocked by washing with a mixture of DCM, MeOH, and DIPEA (17:2:1, respectively). Selective removal of the Fmoc-protecting group was realized by washing three times with 30 mL of a mixture of DMF and piperidine (1:1) to obtain **3**.

In the next step, 16.3 mmol H-Lys(Fmoc)-O*t*Bu was dissolved in 100 mL of dry DMF and stirred with 16.3 mmol 1,1'-carbonyldiimidazol (CDI) and 16.6 mmol DIPEA at RT for 1 h. The glutamate-immobilized resin **3** was added afterward in one portion to the solution of the CDI-adduct of the lysine moiety and stirred for 16 h.

The product **4** was filtered off and washed with DCM and DMF. Selective removal of the Fmoc-protecting group was realized by washing with a mixture of DMF and piperidine (1:1) first for 2 min and then for a second washing for 5 min. The resin-immobilized and *t*Bu-protected binding motif **5** was dried under vacuum.

Classification: Restricted

Linker coupling for P17

Scheme S4: Coupling of the P17 linker moiety via adapted methodology.

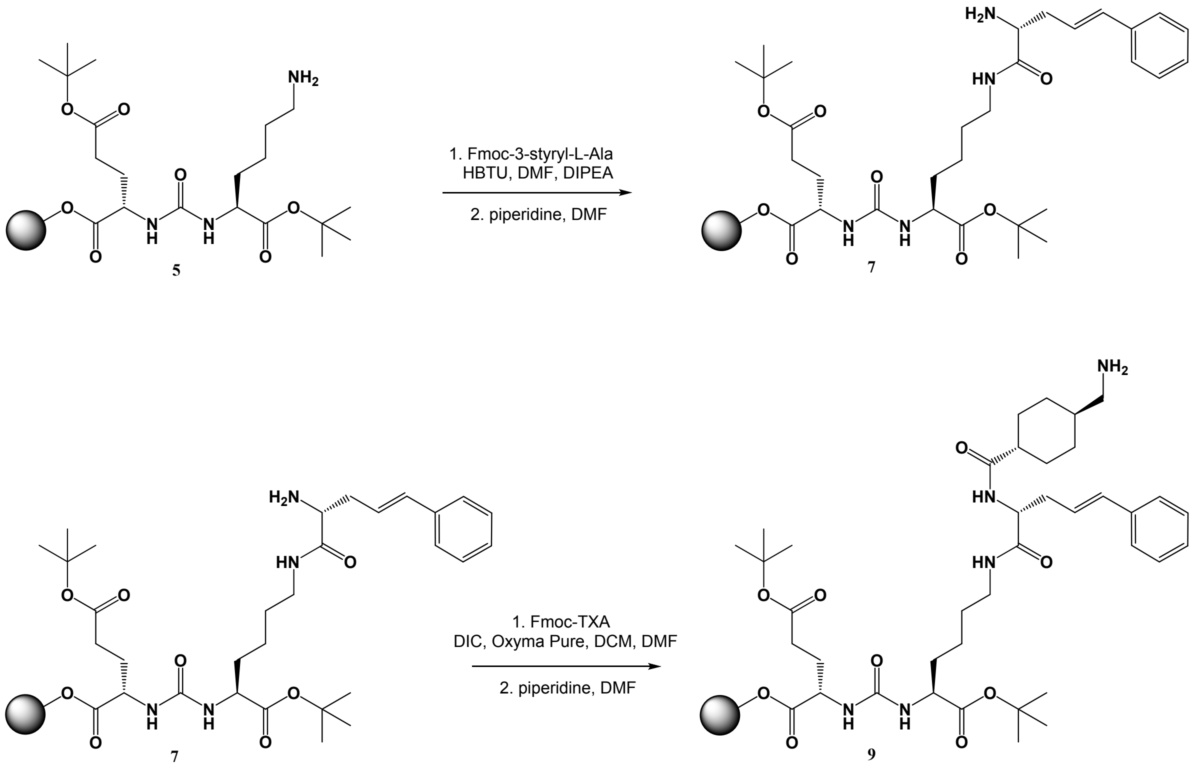

Relative to the resin (0.24 mmol), 4 equiv Fmoc-3-styryl-L-Ala (0.96 mmol) were activated with 3.92 equiv HBTU (*O*-(benzotriazol-1-yl)-*N*,*N*,*N'*,*N'*-tetramethyluronium hexafluorophosphate, 0.94 mmol) in the presence of 4 equiv DIPEA (0.96 mmol) in 3 mL dry DMF. Two min after the addition of DIPEA, the solution was added to the DMF pre-swollen immobilized pharmacophore **5** and agitated at RT for 1 h. Selective removal of the Fmoc-protecting group from the product **6** was realized by washing with a mixture of DMF and piperidine (1:1) first for 2 min and then for a second washing for 5 min to obtain the product **7**.

In the next step, 5 equiv Fmoc-TXA (1.2 mmol) were reacted with 5 equiv Oxyma Pure (1.2 mmol) and 5 equiv DIC (1,1'-carbonyldiimidazol, 1.2 mmol) in 10 mL dry DMF and DCM (1:1). The solution was added to the DMF pre-swollen immobilized pharmacophore **7** and agitated at RT for 1 h. Selective removal of the Fmoc-protecting group from the product **8** was realized by washing with a mixture of DMF and piperidine (1:1) first for 2 min and then for a second washing for 5 min to obtain the product **9**.

Classification: Restricted

Classification: Restricted

Linker coupling for P18

Scheme S5: Coupling of the P18 linker moiety via adapted methodology.

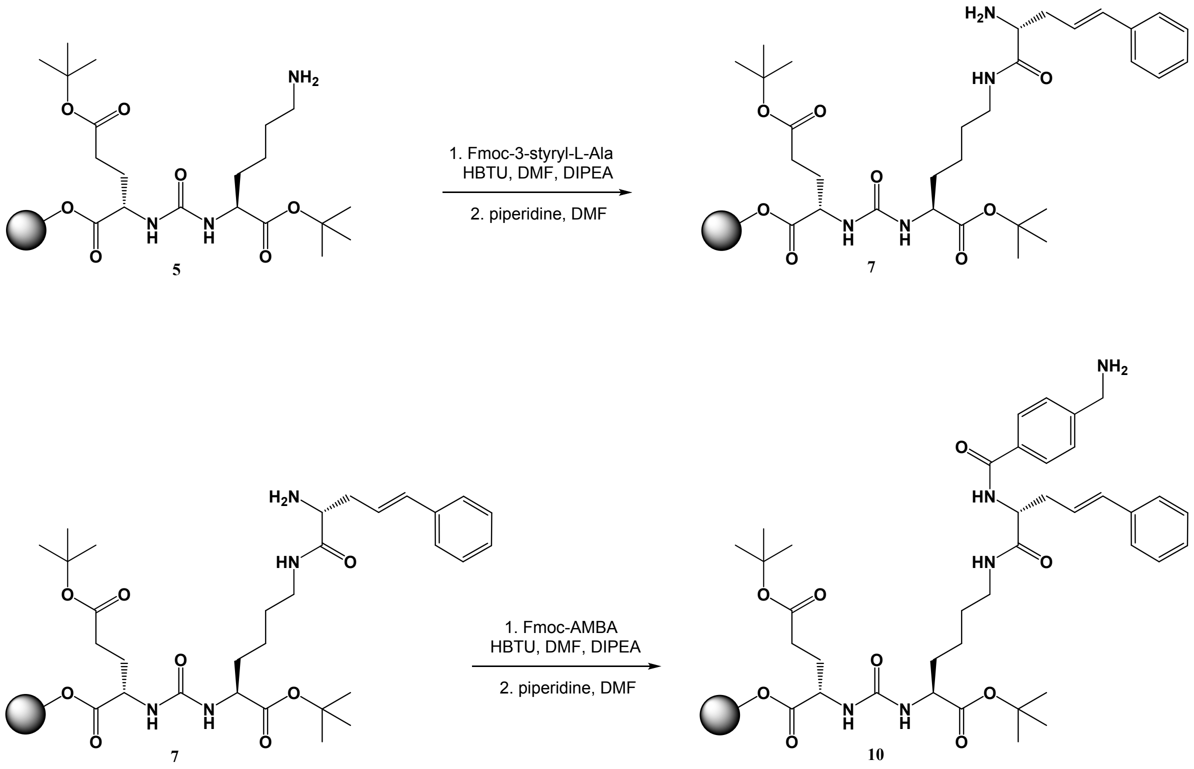

Relative to the resin (0.24 mmol), 4 equiv Fmoc-3-styryl-L-Ala (0.96 mmol) were activated with 3.92 equiv HBTU (0.94 mmol) in the presence of 4 equiv DIPEA (0.96 mmol) in 3 mL dry DMF. Two min after the addition of DIPEA, the solution was added to the DMF pre-swollen immobilized pharmacophore **5** and agitated at RT for 1 h. Selective removal of the Fmoc-protecting group from the product **6** was realized by washing with a mixture of DMF and piperidine (1:1) first for 2 min and then for a second washing for 5 min to obtain the product **7**.

In the next step, 4 equiv Fmoc-AMBA (4-(Fmoc-aminomethyl)benzoic acid, 0.96 mmol) were reacted with 3.92 equiv HBTU (0.94 mmol) and 4 equiv DIPEA (0.96 mmol) in 3 mL dry DMF. Two min after the addition of DIPEA, the solution was added to the DMF pre-swollen immobilized pharmacophore **7** and agitated at RT for 1 h. Selective removal of the Fmoc-protecting group from the product **8** was realized by washing with a mixture of DMF and piperidine (1:1) first for 2 min and then for a second washing for 5 min to obtain the product **10**.

Classification: Restricted

Classification: Restricted

Chelator conjugation for both P17 and P18

Scheme S6: Coupling of the DOTA-*tris*(*t*Bu)ester.

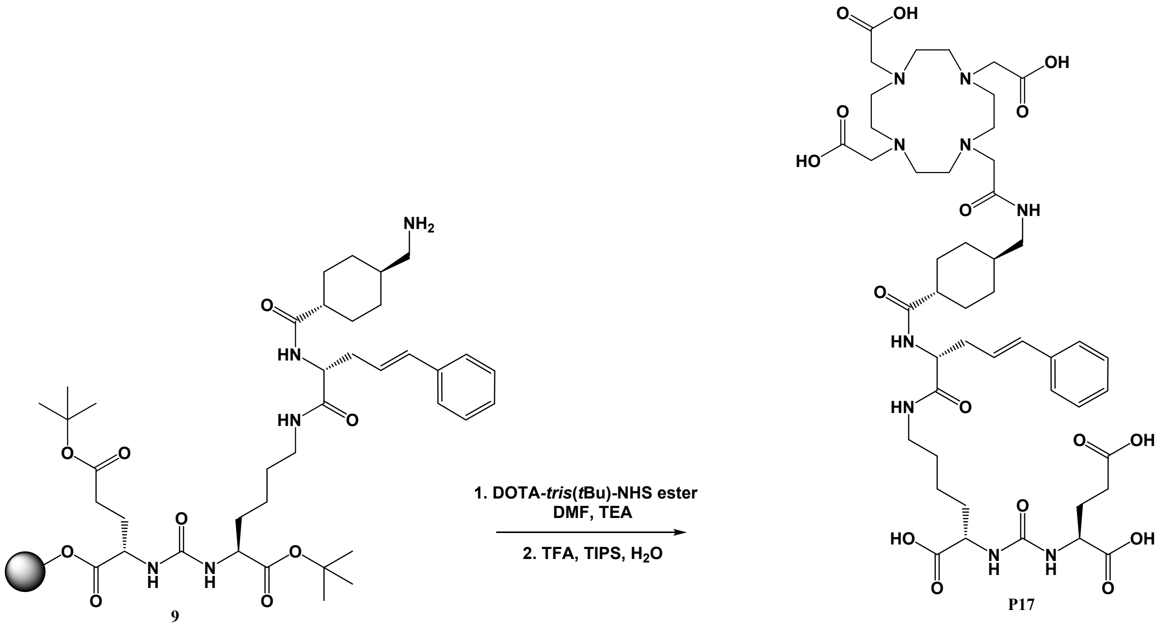

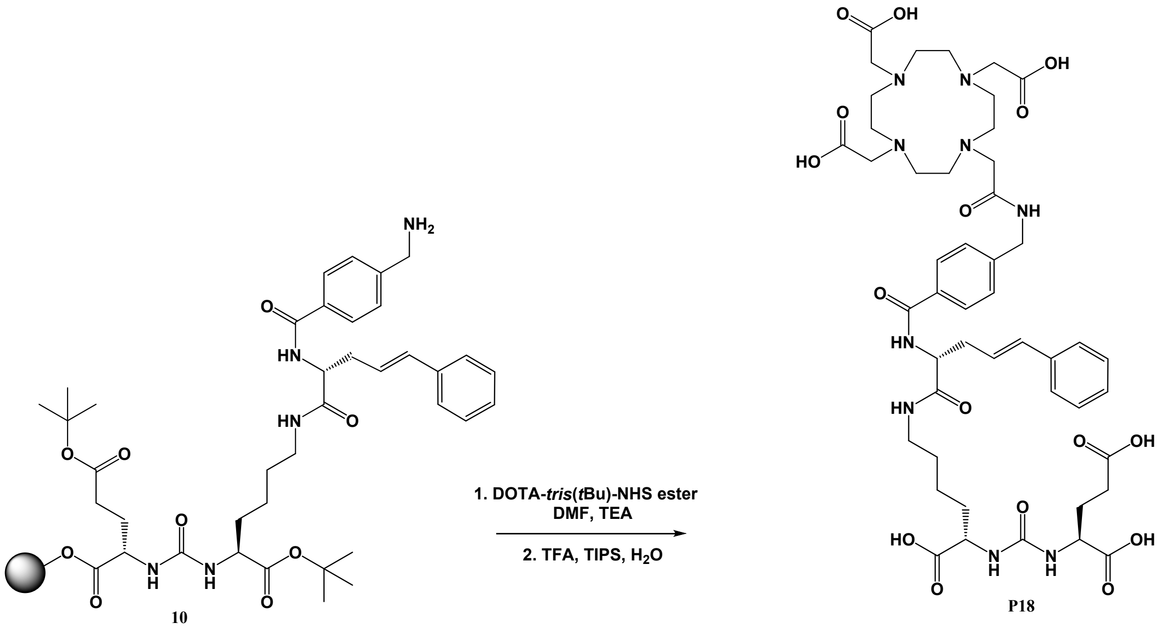

Relative to the resin (0.24 mmol), 4 equiv DOTA-*tris*(*t*Bu)-NHS ester (tri-*tert*-butyl 2,2′,2”-(10-(2-((2,5-dioxopyrrolidin-1-yl)oxy)-2-oxoethyl)-1,4,7,10-tetraazacyclododecane-1,4,7-triyl)triacetate, 0.96 mmol) were dissolved in 3 mL dry DMF in the presence of 4 equiv TEA (0.96 mmol). The solution was added to the DMF pre-swollen immobilized **9** or **10** and agitated at RT for 3 h.

Classification: Restricted

The final products P17 and P18 were obtained by agitation followed by subsequent cleavage from the resin and *t*Bu deprotection after 3 h by applying a mixture of trifluoroacetic acid (TFA), triisopropylsilane (TIPS), and water (95:2.5:2.5, respectively). The solvent mixture was evaporated and the crude product dissolved in acetonitrile and water (1:1) for subsequent purification by RP-HPLC.

Classification: Restricted

Quality control (ESI-MS)

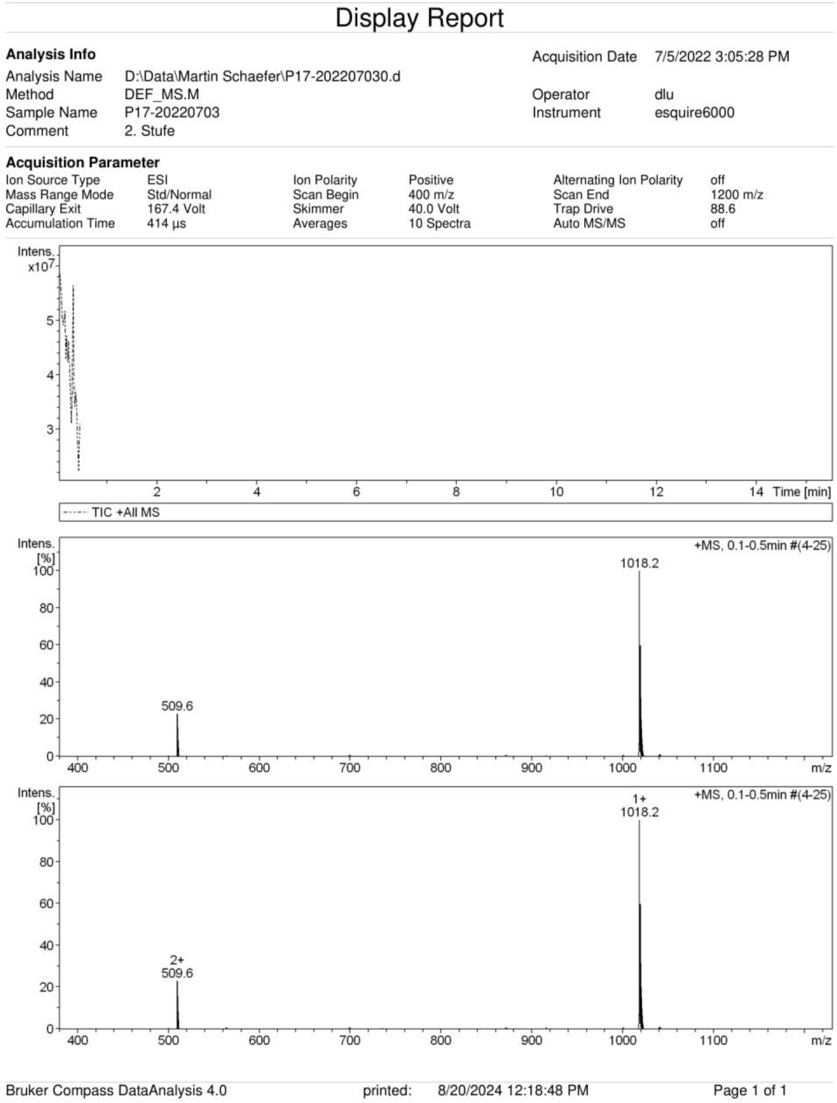

Figure S1: Low resolution ESI-MS of P17. The calculated *m*/*z* for $[M + H^+]^+$ is 1,018.5 g/mol and for $[M + 2H^+]^{+2}$ is 509.8 g/mol.

Classification: Restricted

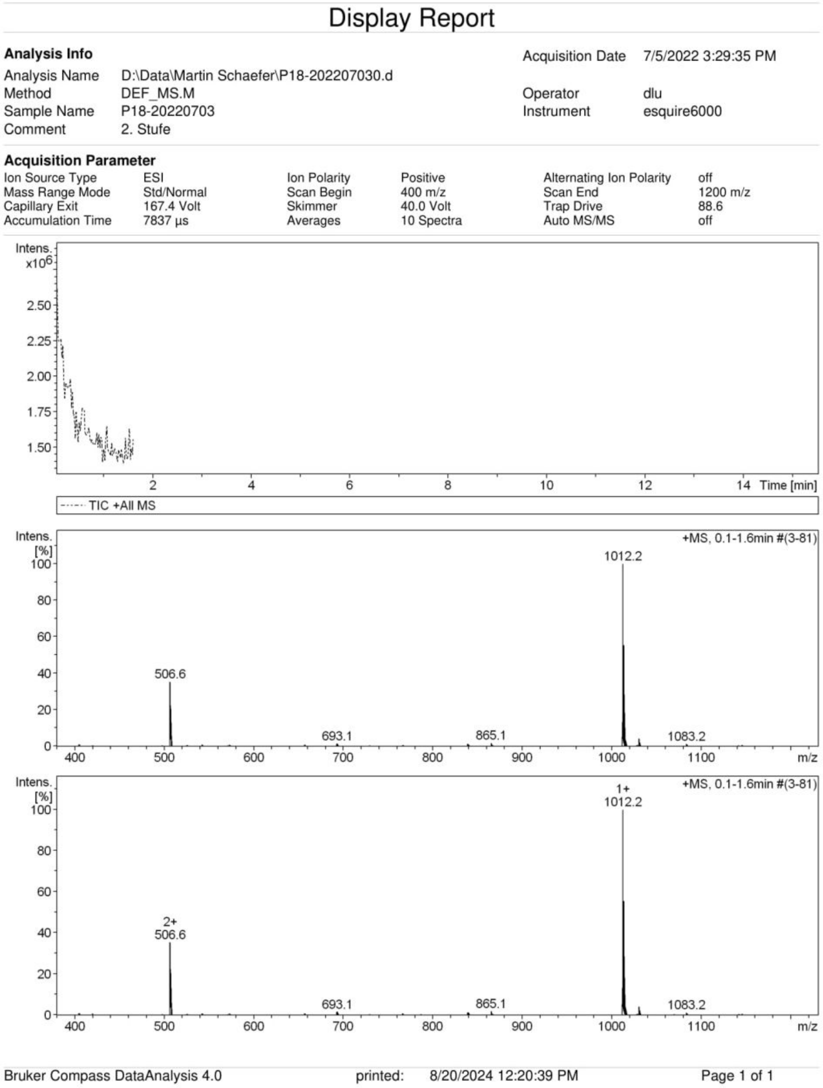

Figure S2: Low resolution ESI-MS of P18. The calculated *m/z* for $[M + H^+]^+$ is 1,012.5 g/mol and for $[M + 2H^+]^{+2}$ is 506.7 g/mol.

Classification: Restricted

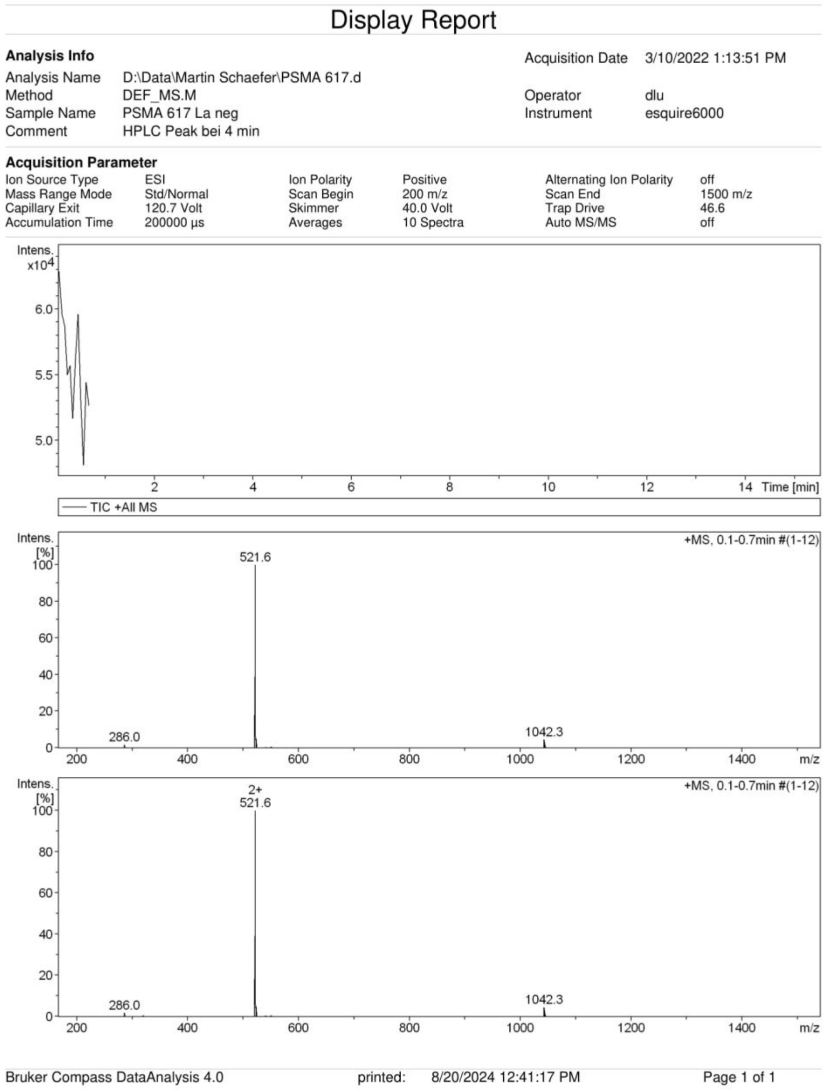

Figure S3: Low resolution ESI-MS of PSMA-617. The calculated *m/z* for $[M + H^+]^+$ is 1,042.5 g/mol and for $[M + 2H^+]^{+2}$ is 521.8 g/mol.

Classification: Restricted

Quality control (MALDI-MS)

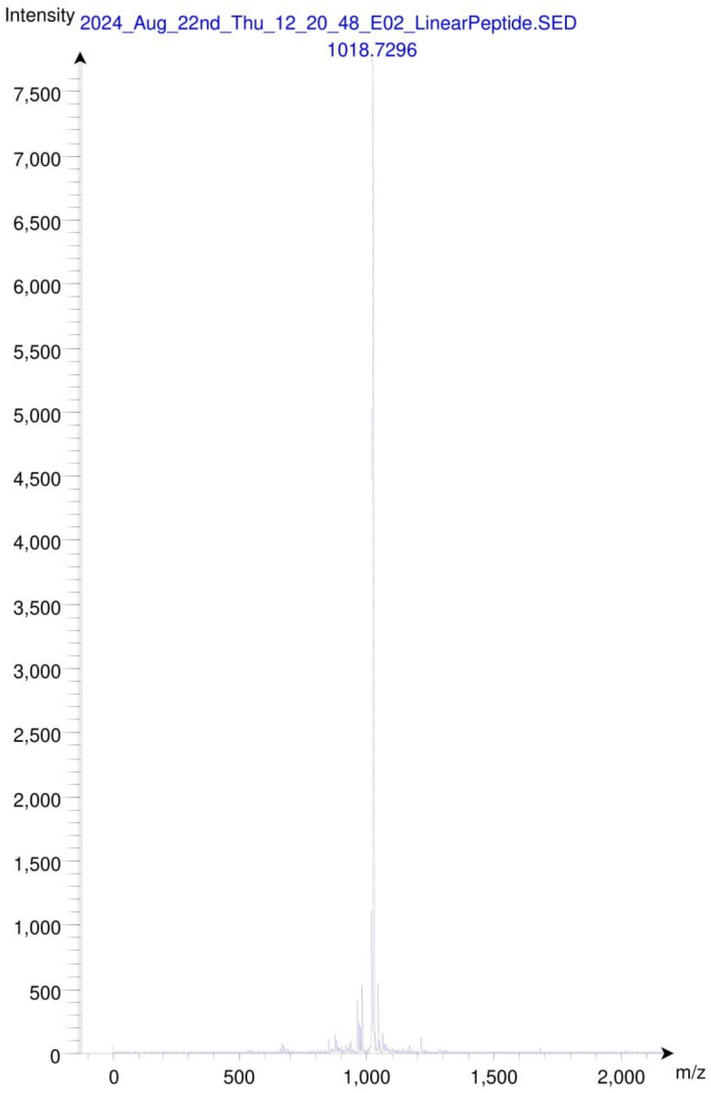

Figure S4: High resolution MALDI-MS of P17. The calculated *m*/*z* for $[M + H^{+}]^{+}$ is 1,018.5097 g/mol.

Classification: Restricted

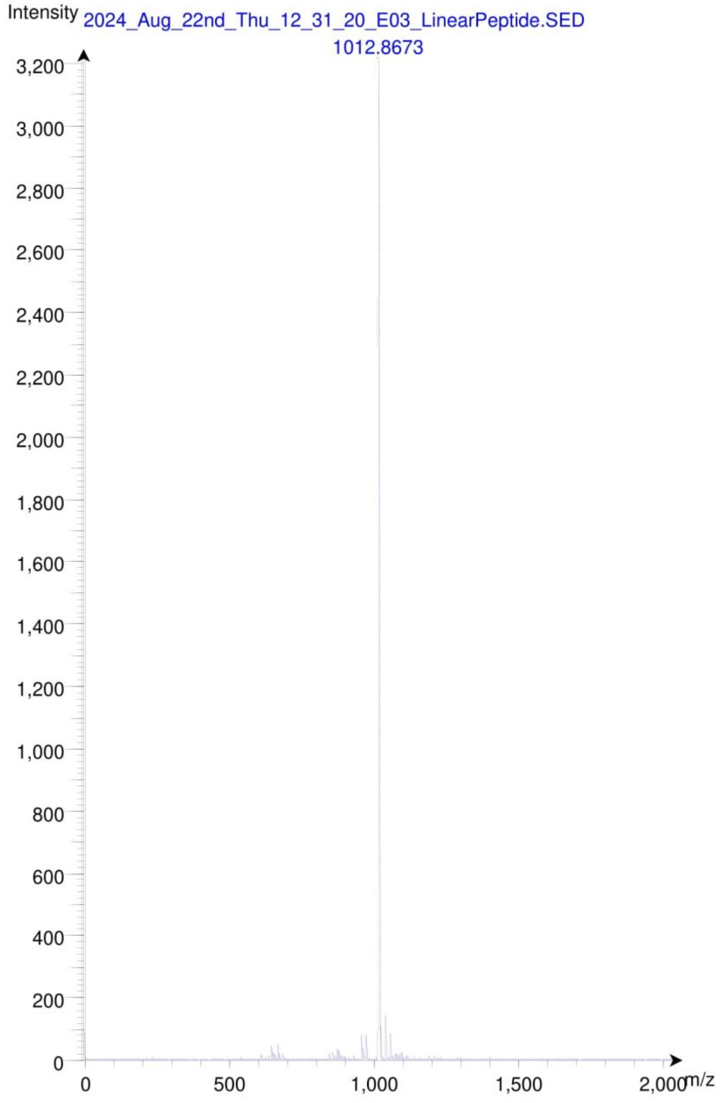

Figure S5: High resolution MALDI-MS of P18. The calculated *m*/*z* for $[M + H^+]^+$ is 1,012.4628 g/mol.

Classification: Restricted

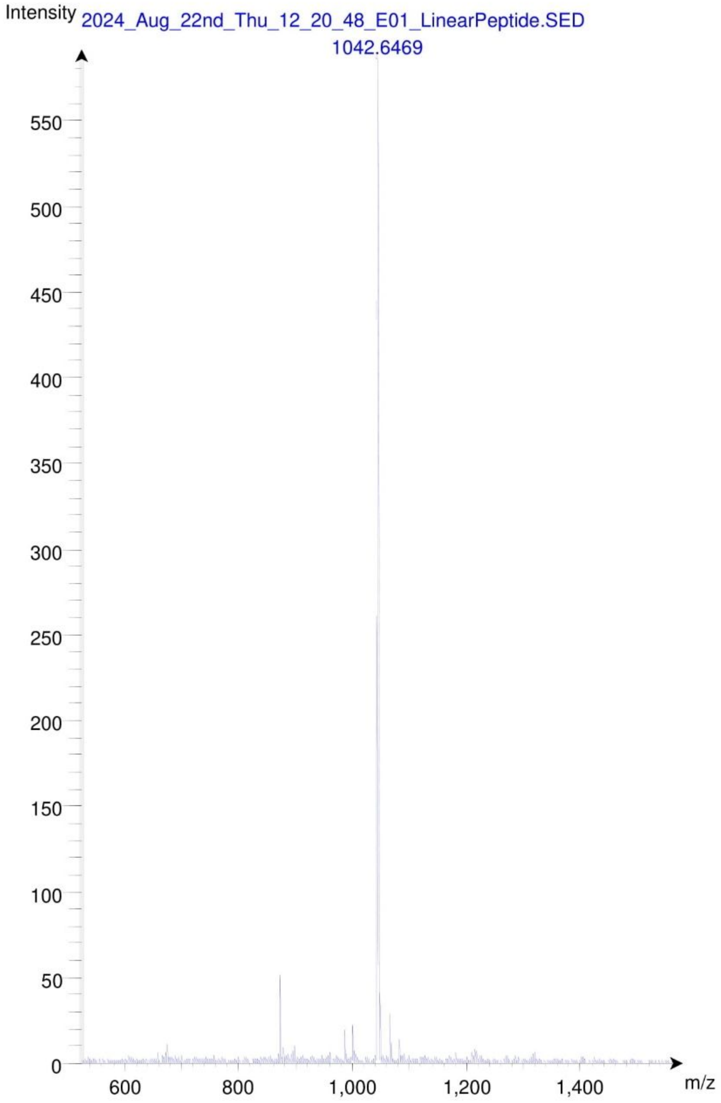

Figure S6: High resolution MALDI-MS of PSMA-617. The calculated *m*/*z* for $[M + H^{+}]^{+}$ is 1,042.5097 g/mol.

Classification: Restricted

Quality control (HPLC)

Chromatogram and Results

Injection Details

Injection Name:	**P17**	*Run Time (min):*	**17,00**
Vial Number:	**RA4**	*Injection Volume:*	**10,00**
Injection Type:	**Unknown**	*Channel:*	**UV_VIS_1**
Calibration Level:		*Wavelength:*	**254**
Instrument Method:	**YR 5_95 ACN_H2O 2ml 15min**	*Bandwidth:*	**n.a.**
Processing Method:	**Martins Processing Method**	*Dilution Factor:*	**1,0000**
Injection Date/Time:	**22.Aug.24 12:19**	*Sample Weight:*	**1,0000**

Chromatogram

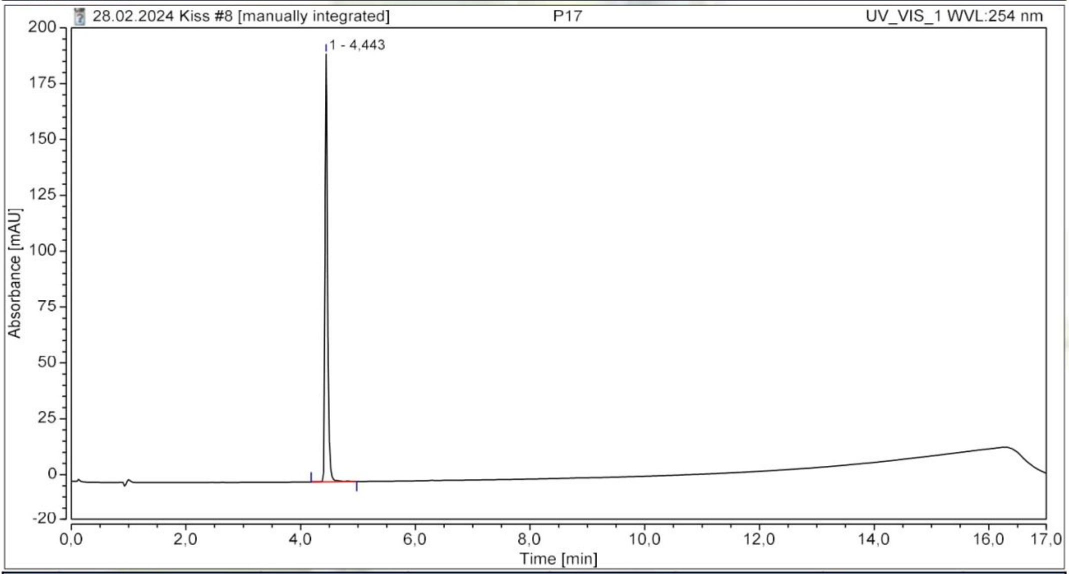

Integration Results

No.	Peak Name	Retention Time min	Area mAU*min	Height mAU	Relative Area %	Relative Height %	Amount
1		4,443	9,609	191,439	100,00	100,00	n.a.
n.a.	Kiss 10	n.a.	n.a.	n.a.	n.a.	n.a.	n.a.
**Total:**			**9,609**	**191,439**	**100,00**	**100,00**	

Default/Integration

© 2009-2020 Thermo Fisher Scientific Inc. All rights reserved.
Chromeleon 7.3.0.60919

Figure S7: HPLC of P17.

Classification: Restricted

Chromatogram and Results

Injection Details

Injection Name:	**P18**	*Run Time (min):*	**17,00**
Vial Number:	**RA3**	*Injection Volume:*	**10,00**
Injection Type:	**Unknown**	*Channel:*	**UV_VIS_1**
Calibration Level:		*Wavelength:*	**254**
Instrument Method:	**YR 5_95 ACN_H2O 2ml 15min**	*Bandwidth:*	**n.a.**
Processing Method:	**Martins Processing Method**	*Dilution Factor:*	**1,0000**
Injection Date/Time:	**22.Aug.24 12:01**	*Sample Weight:*	**1,0000**

Chromatogram

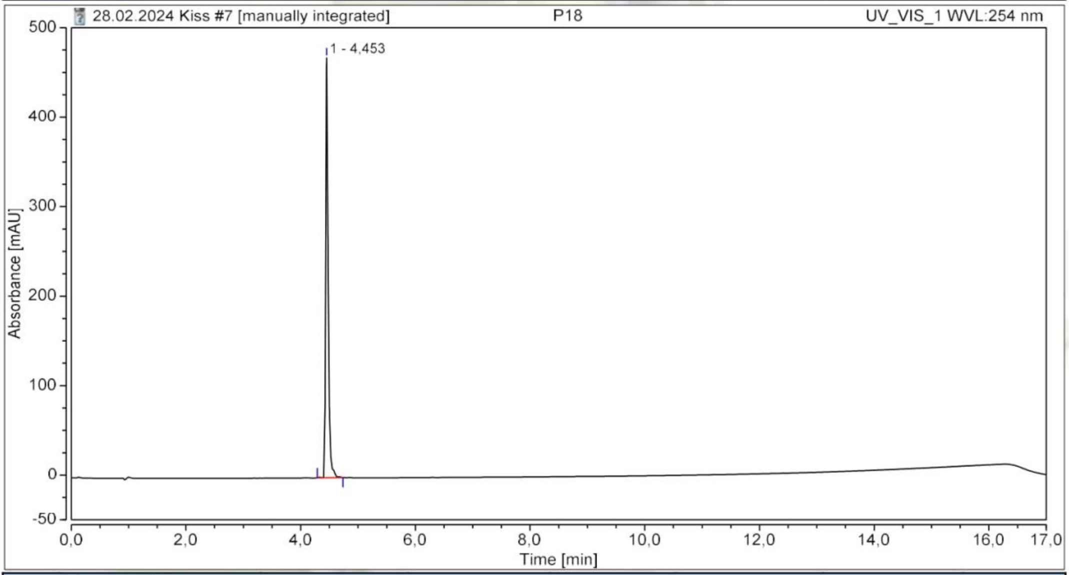

Integration Results

No.	Peak Name	Retention Time min	Area mAU*min	Height mAU	Relative Area %	Relative Height %	Amount
1		4,453	23,856	469,392	100,00	100,00	n.a.
n.a.	Kiss 10	n.a.	n.a.	n.a.	n.a.	n.a.	n.a.
**Total:**			**23,856**	**469,392**	**100,00**	**100,00**	

Default/Integration
© 2009-2020 Thermo Fisher Scientific Inc. All rights reserved.
Chromeleon 7.3.0.60919

Figure S8: HPLC of P18.

Classification: Restricted

Instrument:U3000 Sequence:28.02.2024 Kiss Page 1 of 1

Chromatogram and Results

Injection Details

Injection Name:	**617**	*Run Time (min):*	**17,00**
Vial Number:	**RA2**	*Injection Volume:*	**10,00**
Injection Type:	**Unknown**	*Channel:*	**UV_VIS_1**
Calibration Level:		*Wavelength:*	**254**
Instrument Method:	**YR 5_95 ACN_H2O 2ml 15min**	*Bandwidth:*	**n.a.**
Processing Method:	**Martins Processing Method**	*Dilution Factor:*	**1,0000**
Injection Date/Time:	**22.Aug.24 14:05**	*Sample Weight:*	**1,0000**

Chromatogram

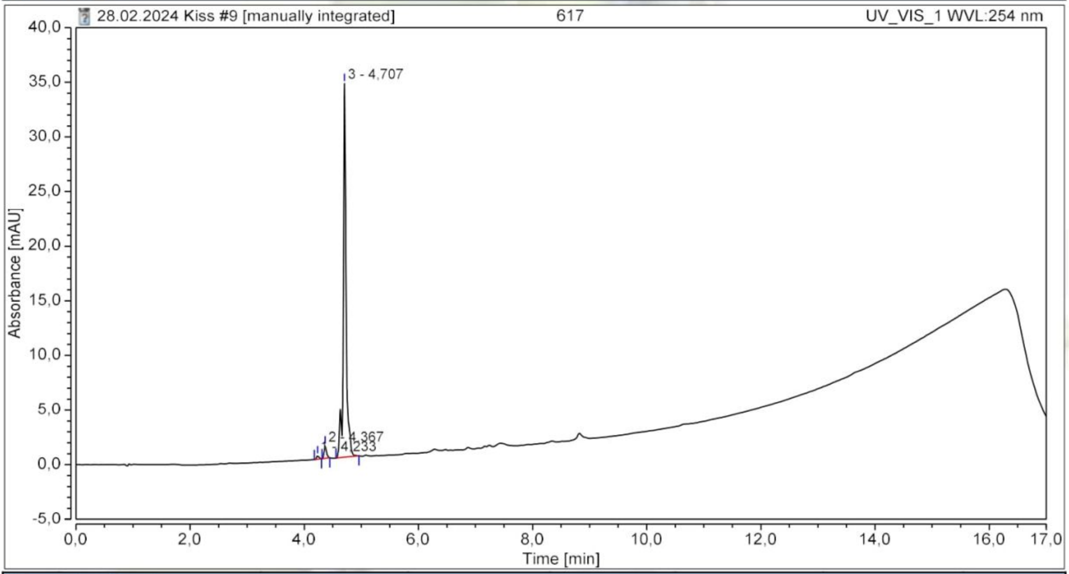

Integration Results

No.	Peak Name	Retention Time min	Area mAU*min	Height mAU	Relative Area %	Relative Height %	Amount
1		4,233	0,015	0,322	0,74	0,90	n.a.
2		4,367	0,053	1,142	2,59	3,20	n.a.
3		4,707	1,993	34,208	96,67	95,89	n.a.
n.a.	Kiss 10	n.a.	n.a.	n.a.	n.a.	n.a.	n.a.
**Total:**			**2,062**	**35,673**	**100,00**	**100,00**	

Default/Integration

© 2009-2020 Thermo Fisher Scientific Inc. All rights reserved.
Chromeleon 7.3.0.60919

Figure S9: HPLC of PSMA-617.

Classification: Restricted

NMR data for P17 and P18

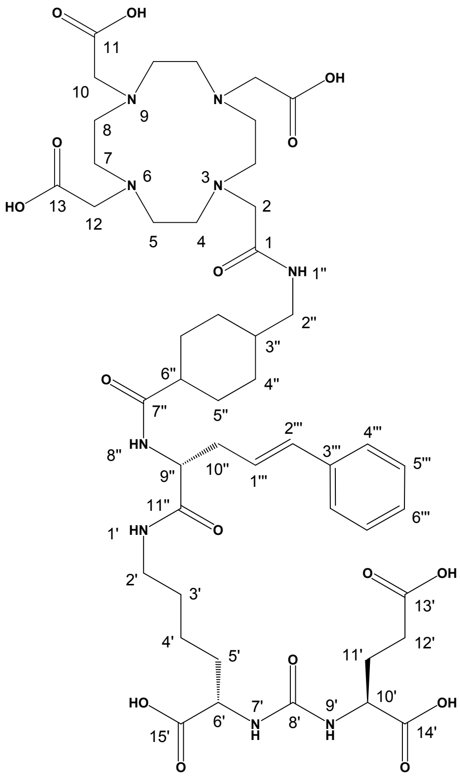

P17

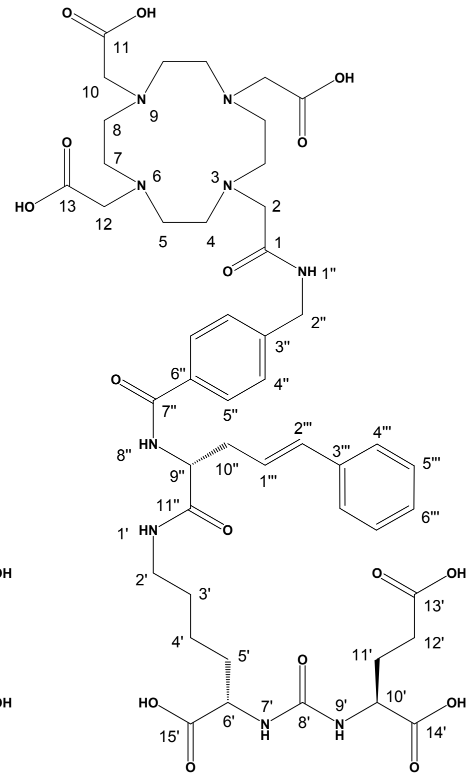

P18

NMR data for P17

^{1}H NMR: 8.434 (v br s, H-1''), 7.930 (t, $J_{H2'}$ = 5.63 Hz, H-1'), 7.828 (d, $J_{H9''}$ = 8.34 Hz, H-8''), 7.33–7.28 (4H, ol 2 × ho m, H-4''' and H-5'''), 7.201 (ho "t", H-6'''), 6.390 (dt, $J_{H1'''}$ = 15.77, $J_{H10a''}$ = $J_{H10b''}$ = 1.3 Hz, H-2'''), 6.329 (d, $J_{H10'}$ = 8.26 Hz, H-9'), 6.288 (d, $J_{H6'}$ = 8.26 Hz, H-7'), 6.128 (dt, $J_{H2'''}$ = 15.80, $J_{H10a''}$ = $J_{H10b''}$ = 7.15 Hz, H-1'''), 4.337 (td, $J_{H8''}$ = $J_{H10b''}$ = 8.19, $J_{H10a''}$ = 5.80 Hz, H-9''), 4.096 (td, $J_{H9'}$ = $J_{H11a'}$ = 8.27, $J_{H11b'}$ = 5.26 Hz, H-10'), 4.04 (2H, v br s, H-2 or H-10), 4.013 (td, $J_{H7'}$ = $J_{H5a'}$ = 8.12, $J_{H5b'}$ = 5.3 Hz, H-6'), 3.88 (2H, v br s, H-2 or H-10), 3.56 (4H, v br s, H-12), 3.33 (4H, v br s, H-4 or H-5 or H-7 or H-8), 3.26 (4H, v br s, H-4 or H-5 or H-7 or H-8), 3.07 (4H, v br s, H-4 or H-5 or H-7 or H-8; 3.07 and 3.06 are H-4 and H-5 or H-7 and H-8), 3.065 (ol m, H-2a'), 3.06 (4H, v br s, H-4 or H-5 or H-7 or H-8; 3.07 and 3.06 are H-4 and H-5 or H-7 and H-8), 2.96 (2H, ol m, H-2''), 2.95 (ol m, H-2b'), 2.511 (ho m, $J_{H9''}$ = 5.61 Hz, $J_{H10b''}$ and $J_{H1'''}$ nm, H-10a''), 2.403 (ho m, $J_{H10a''}$ = −13.85, $J_{H9''}$ = 8.18 Hz, $J_{H1'''}$ nm, H-10b''), 2.258 (d_{AB}dd, $J_{H12b'}$ = −16.61, J_{med} = 9.29, J_{sml} = 6.70 Hz, H-12a'), 2.220 (d_{AB}dd, $J_{H12a'}$ = −16.63, J_{med} = 9.30, J_{sml} = 5.79 Hz, H-12b'), 2.158 (br tt, $J_{H5aax''}$ = $J_{H5bax''}$ = 12.0, $J_{H5aeq''}$ = $J_{H5beq''}$ = 3.1 Hz, H-6''), 1.917 (ho m, H-11a'), 1.73 (ol m, H-4aeq'', attached to C-4b''), 1.72 (ol m, H-5aeq'', attached to C-5b''), 1.71 (ol ho m, H-11b'), 1.69 (ol m, H-4beq'', attached to C-4a''), 1.69 (ol m, H-5beq'', attached

Classification: Restricted

to C-5a''), 1.59 (ho m, H-5a'), 1.46 (ho m, H-5b'), 1.35 (2H, ol ho m, H-3'), 1.33 (ol m, H-3''), 1.30 (ol m, H-5cax'', attached to C-5b''), 1.25 (2H, ol ho m, H-4'), 1.25 (ho m, H-5axd'', attached to C-5a''), 0.86 (ol m, H-4cax'', attached to C-4b''), 0.85 (ol m, H-4dax'', attached to C-4a''). Exchangeable H's various broad signals not assigned.

^{13}C NMR: 174.99 (C-7''), 174.55 (C-15'), 174.16 (C-14'), 173.72 (C-13'), 171.80 (br, C-13), 170.81 (C-11''), 168.79 (v br, C-11), 165.39 (v br, C-1), 157.29 (C-8'), 137.08 (C-3'''), 131.74 (C-2'''), 128.54 (C-5'''), 127.12 (C-6'''), 126.16 (C-1'''), 125.87 (C-4'''), 54.83 (br, C-2 or C-10), 54.02 (br, C-2 or C-10), 52.63 (v br, C-12), 52.26 (C-6'), 52.06 (C-9''), 51.66 (C-10'), 50.75 (v br, C-4 or C-5 or C-7 or C-8), 50.61 (v br, C-4 or C-5 or C-7 or C-8), 48.38 (v br, C-4 or C-5 or C-7 or C-8; 48.38 and 48.1 are C-4 and C-5 or C-7 and C-8), 48.1 (v br, C-4 or C-5 or C-7 or C-8; 48.38 and 48.1 are C-4 and C-5 or C-7 and C-8), 45.06 (br, C-2''), 43.57 (C-6''), 38.32 (C-2'), 36.78 (C-3''), 35.98 (C-10''), 31.71 (C-5'), 29.89 (C-12'), 29.60 (br, C-4a''), 29.45 (br, C-4b''), 28.89 (br, C-5a''), 28.83 (C-3'), 28.36 (br, C-5b''), 27.54 (C-11'), 22.59 (C-4').

^{15}N NMR: −261.98 (N-8''), −263.64 (N-1''), −265.21 (N-1'), −293.20 (N-7'), −294.26 (N-9').

NMR data for P18

^{1}H NMR: 8.945 (v br s, H-1''), 8.414 (d, $J_{H9''}$ = 8.07 Hz, H-8''), 8.063 (t, $J_{H2'}$ = 5.68 Hz, H-1'), 7.859 (2H, AA' part of AA'MM' system, $J_{AM + JAM'}$ = 8.38 Hz, H-5''), 7.369 (2H, br MM' part of AA'MM' system, $J_{AM + JAM'}$ = 8.32 Hz, H-4''), 7.322 (2H, ho "d", H-4'''), 7.284 (2H, ho "t", H-5'''), 7.190 (ho "t", H-6'''), 6.465 (br d, $J_{H1'''}$ = 15.78, Hz, H-2'''), 6.335 (d, $J_{H10'}$ = 8.27 Hz, H-9'), 6.300 (d, $J_{H6'}$ = 8.24 Hz, H-7'), 6.228 (dt, $J_{H2'''}$ = 15.82, $J_{H10a''}$ = $J_{H10b''}$ = 7.14 Hz, H-1'''), 4.553 (td, $J_{H8''}$ = $J_{H10b''}$ = 8.28, $J_{H10a''}$ = 5.81 Hz, H-9''), 4.384 (2H, d, $J_{H1''}$ = 5.64 Hz, H-2''), 4.098 (td, $J_{H9'}$ = $J_{H11a'}$ = 8.28, $J_{H11b'}$ = 5.25 Hz, H-10'), 4.021 (td, $J_{H7'}$ = $J_{H5a'}$ = 8.18, $J_{H5b'}$ = 5.22 Hz, H-6'), 4.00 (2H, v br s, H-2 or H-10), 3.93 (2H, v br s, H-2 or H-10), 3.62 (4H, v br s, H-12), 3.31 (8H, v br s, H-4 and H-5 or H-7 and H-8), 3.11 (8H, v br s, H-4 and H-5 or H-7 and H-8), 3.099 (ho m, H-2a'), 2.985 (ho m, H-2b'), 2.669 (ho m, $J_{H10b''}$ = −14.22, $J_{H1'''}$ = 7.32, $J_{H9''}$ = 5.83 Hz, H-10a''), 2.628 (ho m, $J_{H10a''}$ = −14.14, $J_{H9''}$ = 8.65, $J_{H1'''}$ = 7.02 Hz, H-10b''), 2.258 (d$_{AB}$dd, $J_{H12b'}$ = −16.61, J_{med} = 9.29, J_{sml} = 6.70 Hz, H-12a'), 2.219 (d$_{AB}$dd, $J_{H12a'}$ = −16.64, J_{med} = 9.30, J_{sml} = 5.75 Hz, H-12b'), 1.916 (ho m, H-11a'), 1.707 (ho m, H-11b'), 1.605 (ho m, H-5a'), 1.467 (ho m, H-5b'), 1.375 (2H, ho m, H-3'), 1.268 (2H, ho m, H-4'). Exchangeable H's various broad signals but not assigned.

^{13}C NMR: 174.56 (C-15'), 174.16 (C-14'), 173.72 (C-13'), 171.57 (br, C-13), 170.83 (C-11''), 169.2 (v br, C-11), 166.1 (v br, C-1), 165.97 (C-7''), 157.30 (C-8'), 141.82 (br, C-3''), 137.05 (C-3'''), 132.95 (C-6''), 131.81 (C-2'''), 128.56 (C-5'''), 127.66 (C-5''), 127.15 (C-4''), 127.15 (C-6'''), 126.42 (C-1'''), 125.89 (C-4'''), 54.68 (br, C-2 or C-10), 53.92 (br, C-2 or C-10), 53.28 (br, C-9''), 52.88 (v br, C-12), 52.27 (C-6'), 51.66 (C-10'), 50.53 (v br, C-4 or C-5 or C-7 or C-8), 50.4 (v br, C-4 or C-5 or C-7 or C-8), 48.65 (v br, C-4 or C-5 or C-7 or C-8; 48.65 and 48.5 are C-4 and C-5 or C-7 and C-8), 48.5 (v br, C-4 or C-5 or C-7 or C-8; 48.65 and 48.5 are C-4 and C-5 or C-7 and C-8), 42.06 (C-2''), 38.46 (C-2'), 35.53 (C-10''), 31.74 (C-5'), 29.90 (C-12'), 28.85 (C-3'), 27.54 (C-11'), 22.63 (C-4').

Classification: Restricted

^{15}N NMR: −262.56 (N-1''), −264.65 (N-8''), −265.21 (N-1'), −293.20 (N-7'), −294.24 (N-9').

Classification: Restricted

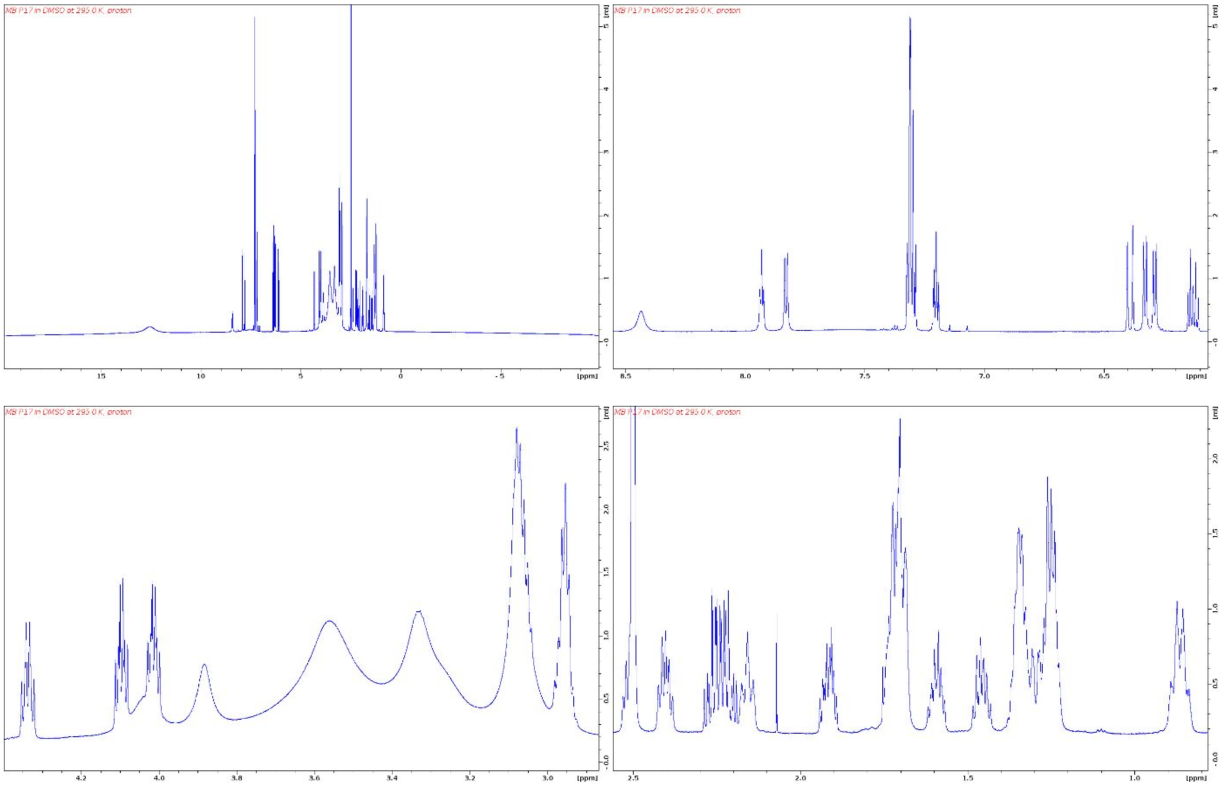

Figure S10: ^{1}H NMR spectrum of P17.

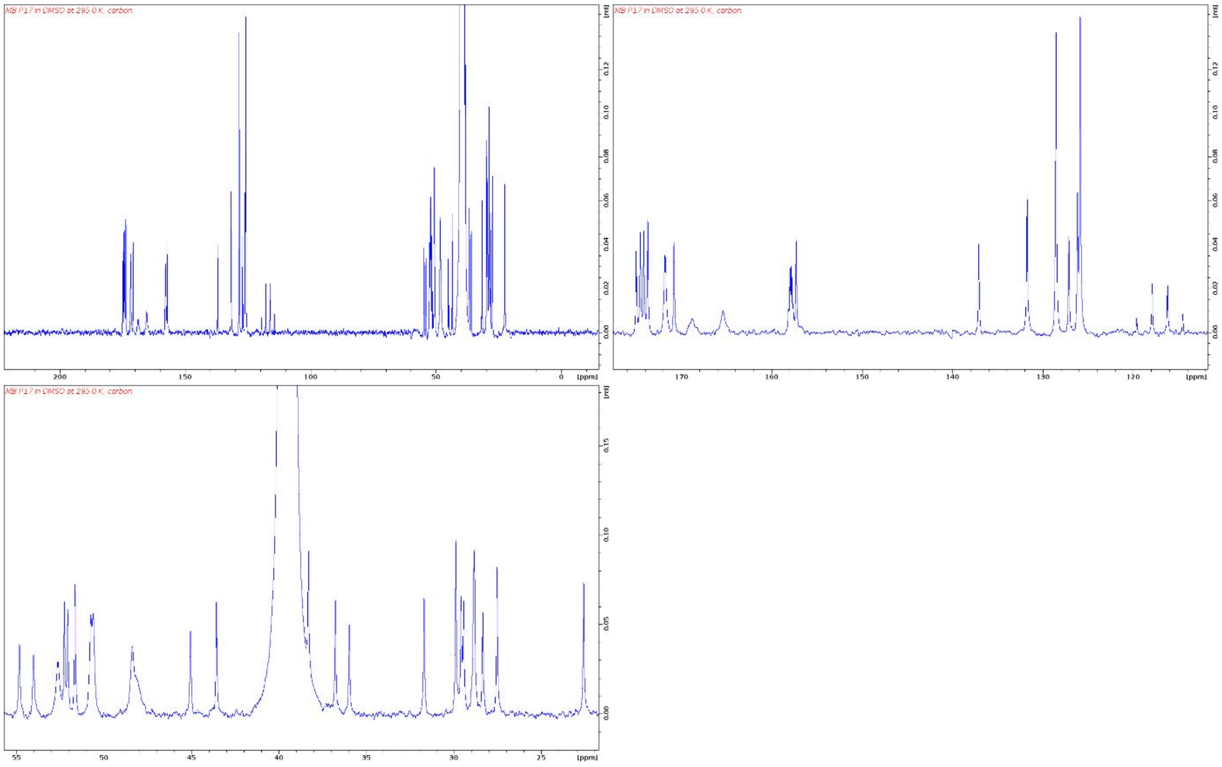

Figure S11: ^{13}C NMR spectrum of P17.

Classification: Restricted

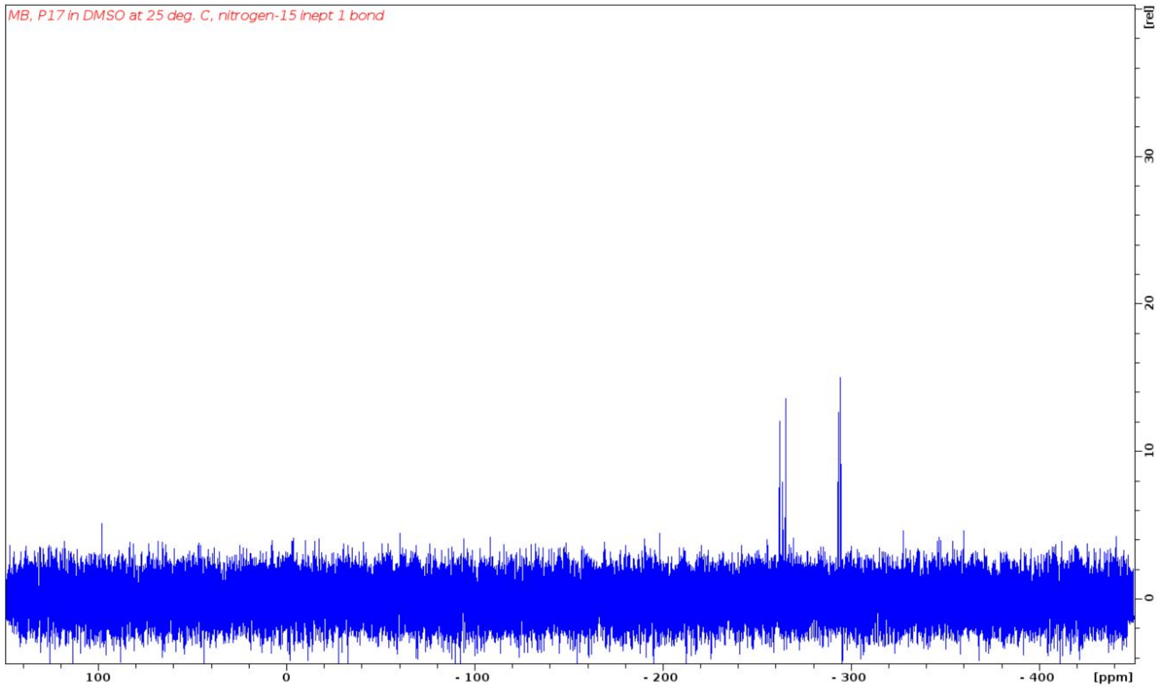

Figure S12: ^{15}N NMR spectrum of P17.

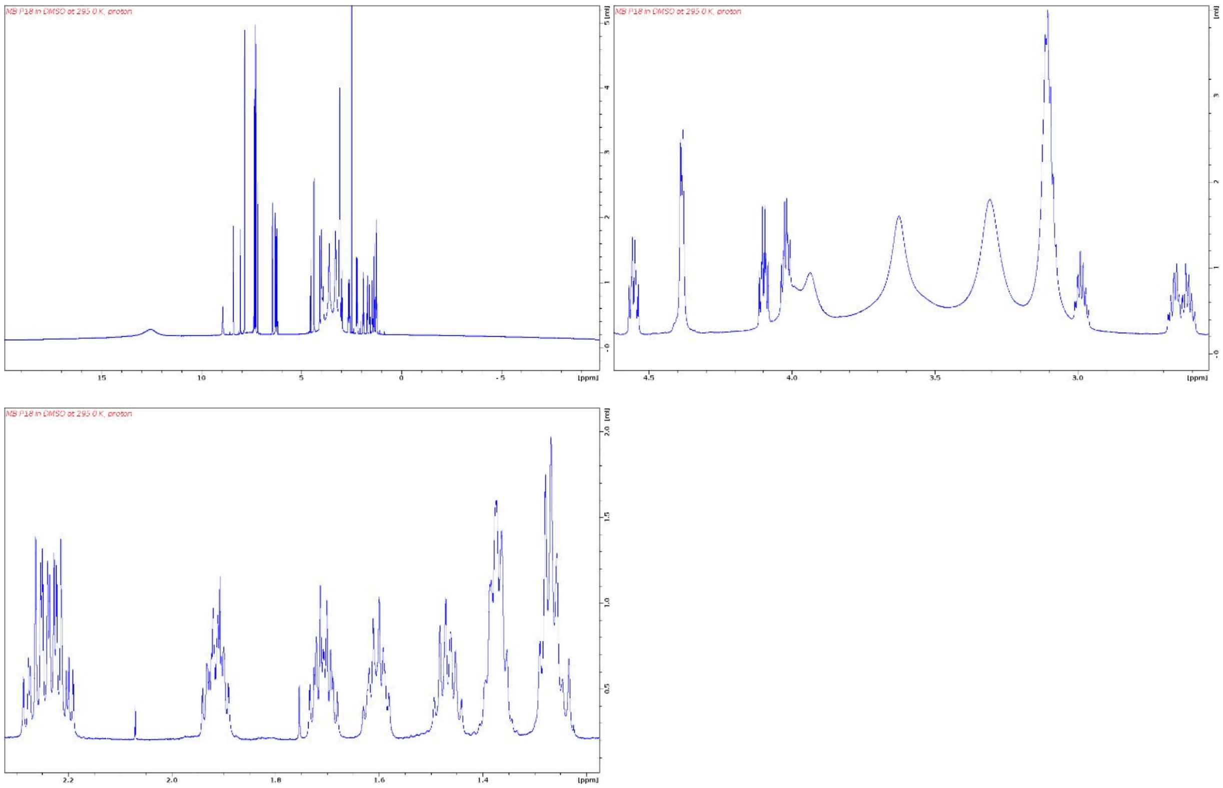

Figure S13: ^{1}H NMR spectrum of P18.

Classification: Restricted

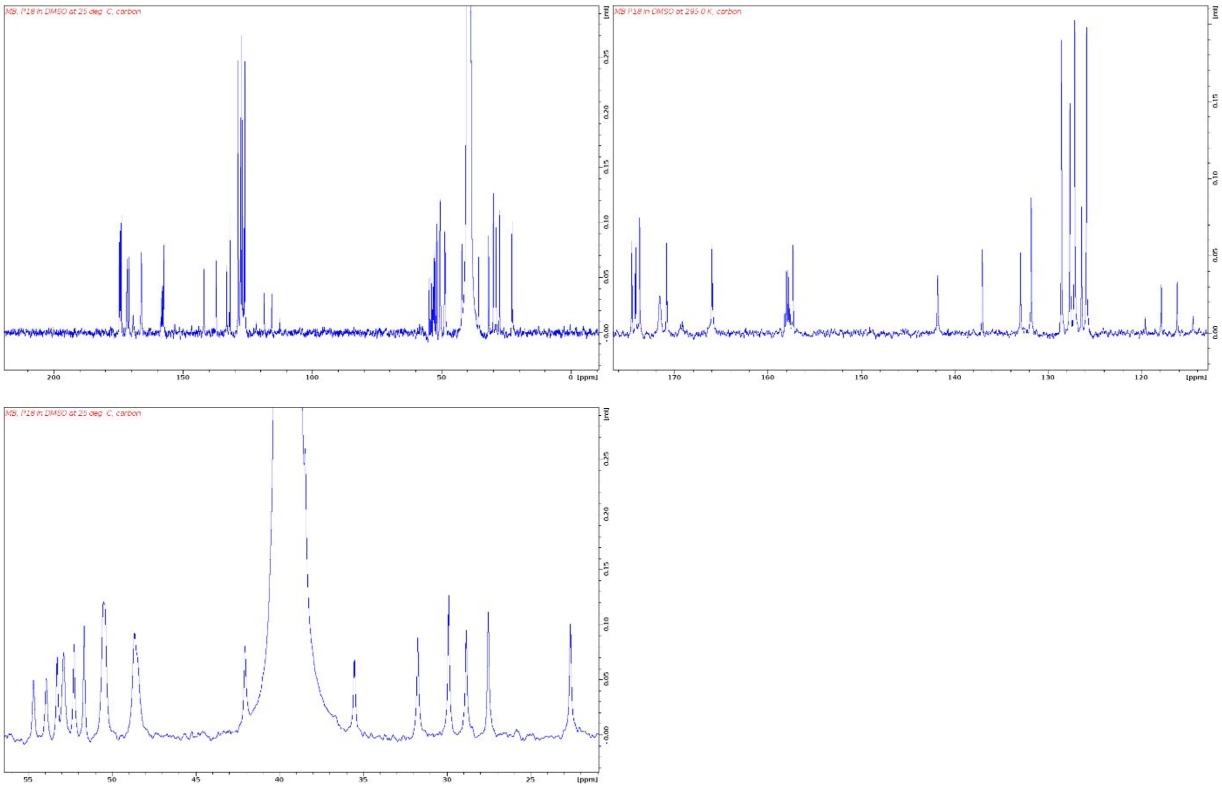

Figure S14: ^{13}C NMR spectrum of P18.

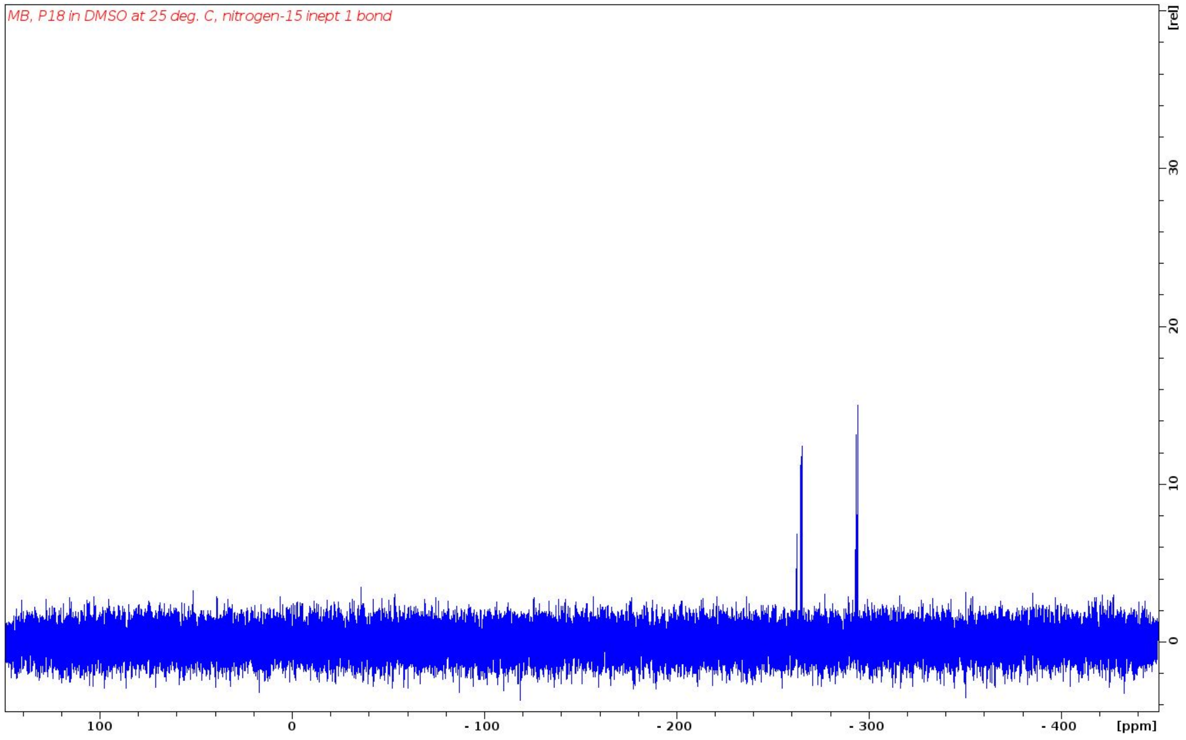

Figure S15: ^{15}N NMR spectrum of P18.

Classification: Restricted

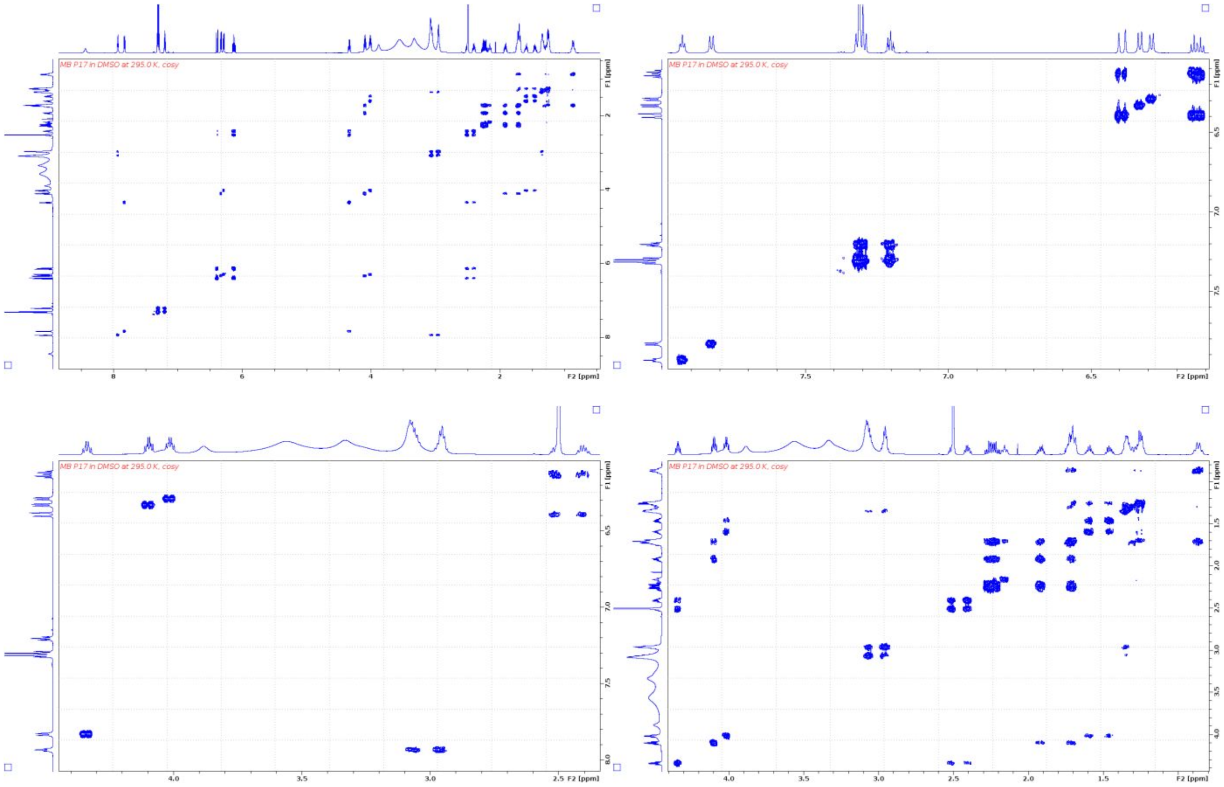

Figure S16: COSY NMR spectrum of P17.

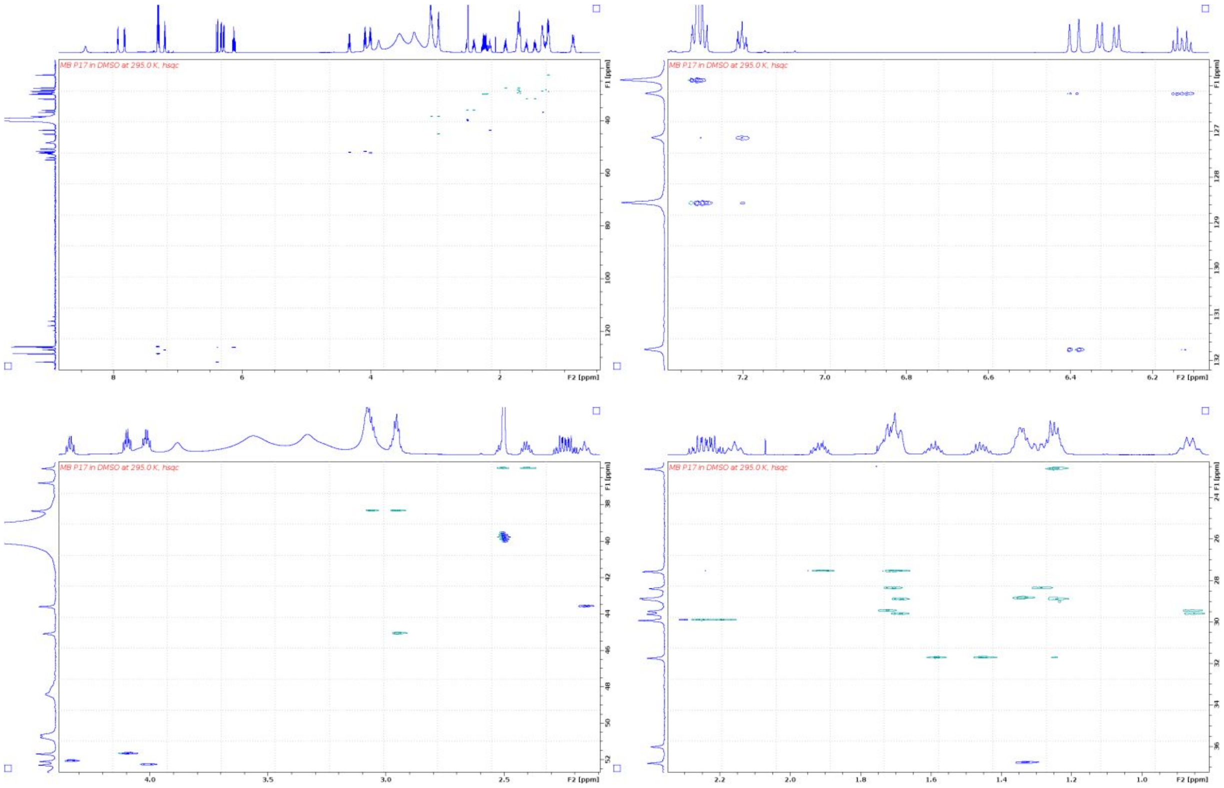

Figure S17: HSQC NMR spectrum of P17.

Classification: Restricted

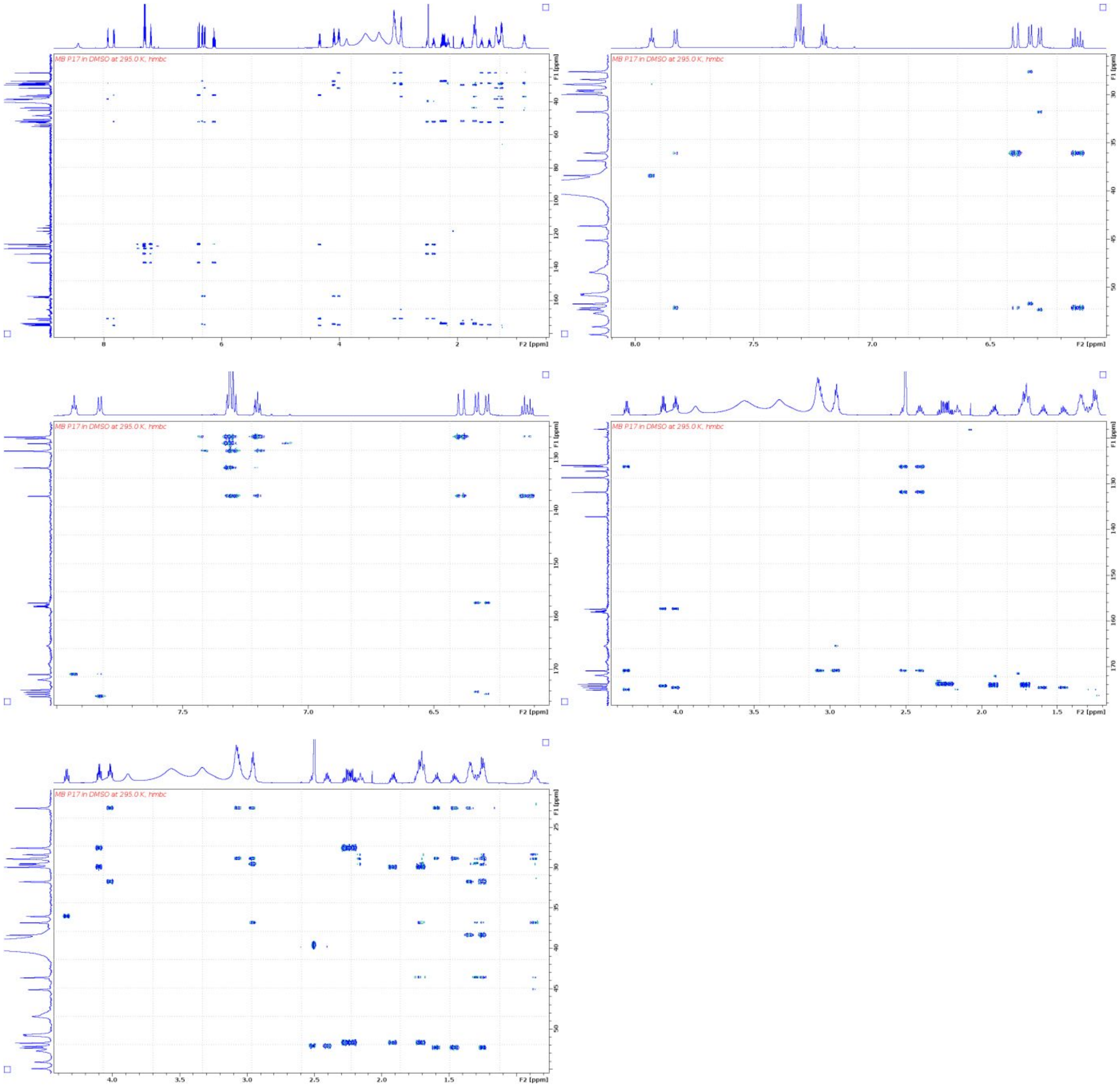

Figure S18: HMBC NMR spectrum of P17.

Classification: Restricted

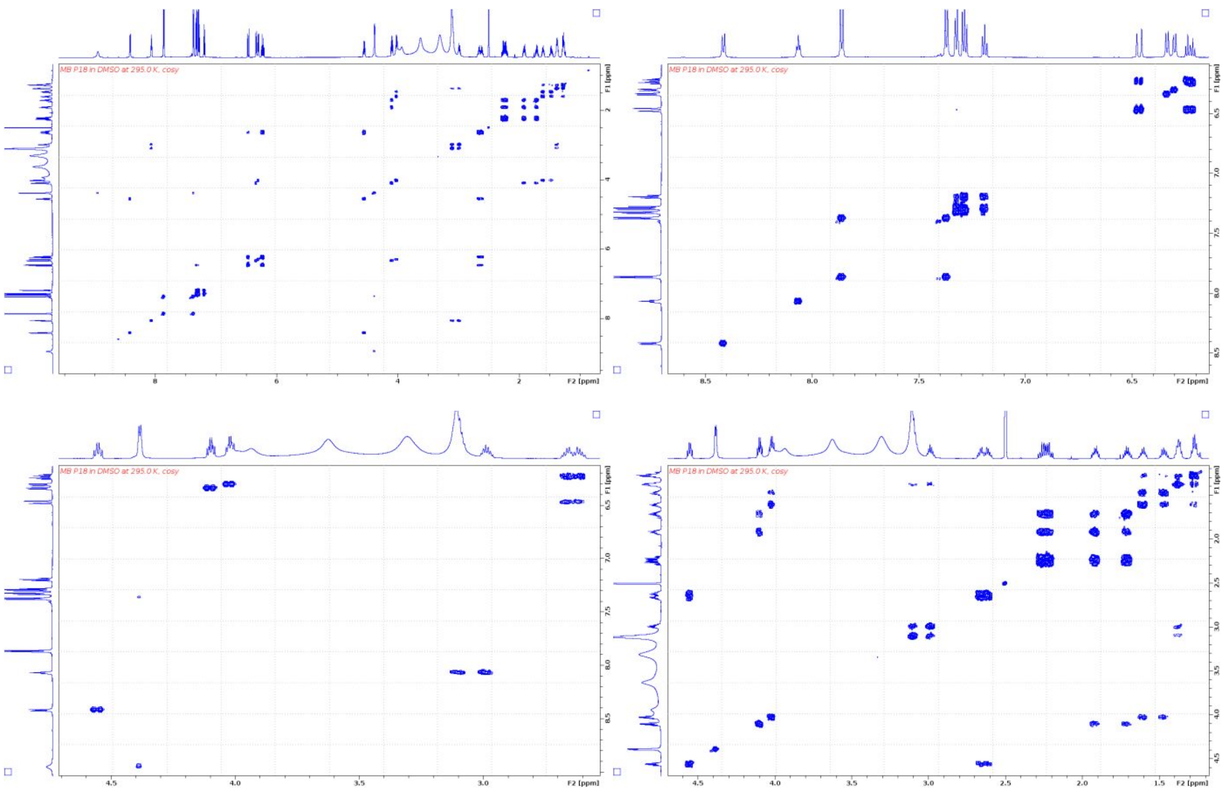

Figure S19: COSY NMR spectrum of P18.

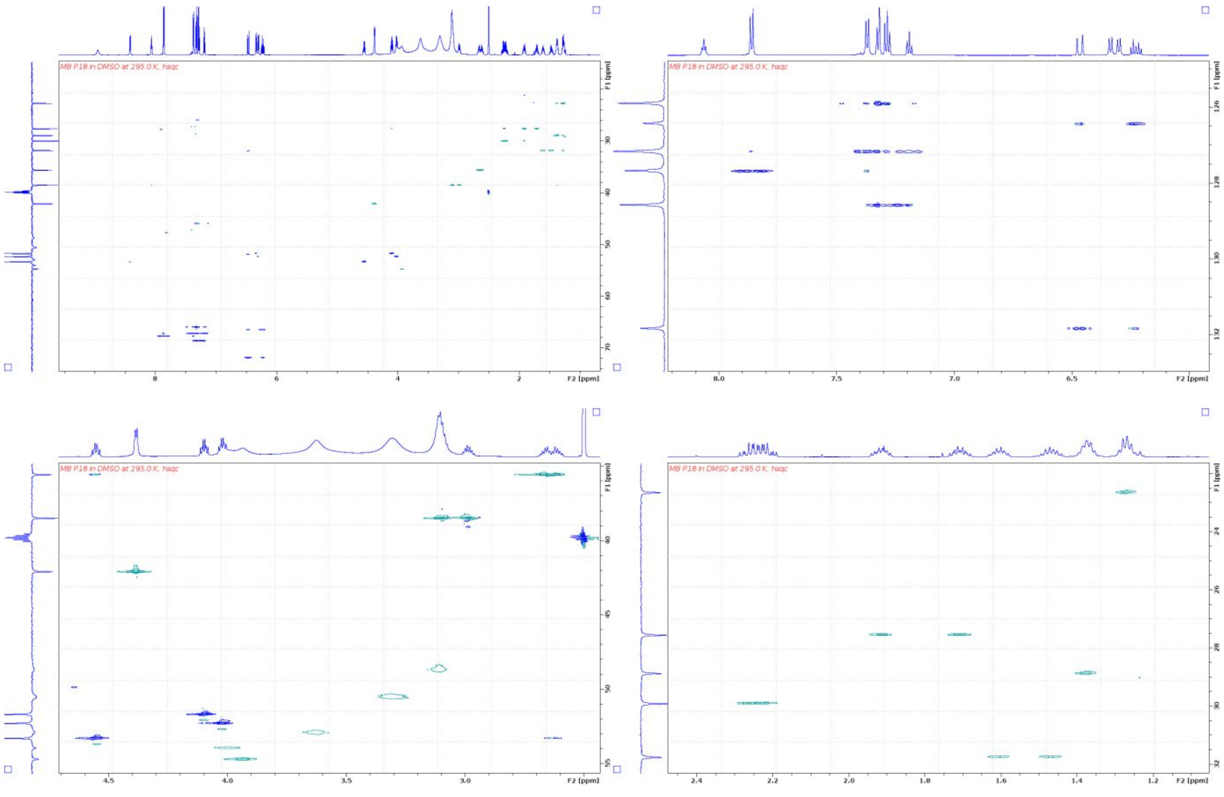

Figure S20: HSQC NMR spectrum of P18.

Classification: Restricted

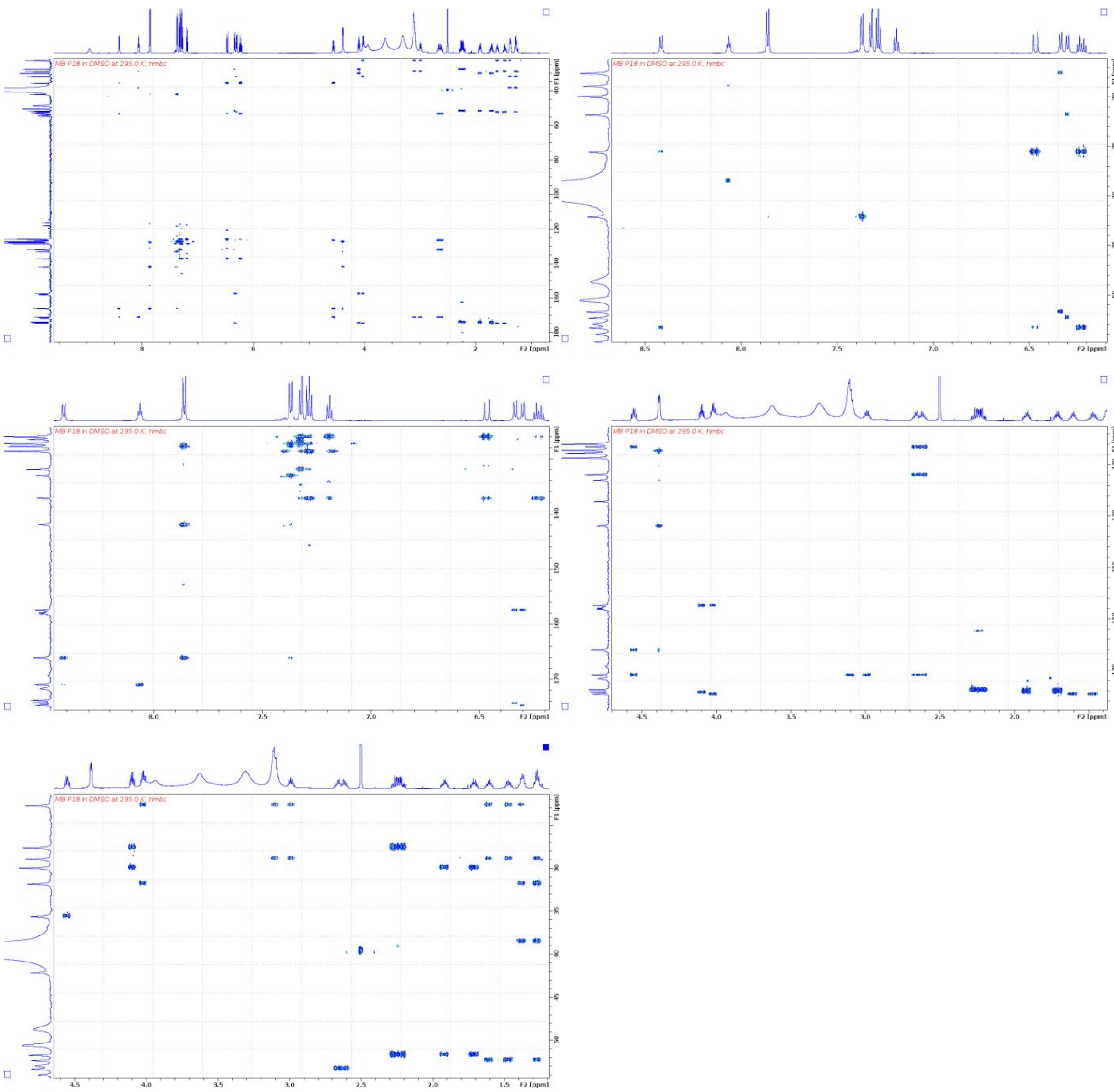

Figure S21: HMBC NMR spectrum of P18.

Classification: Restricted

Quality control (Radio-HPLC)

Instrument:U3000 Sequence:27.08.2024 P18, P17, 617 Radio Page 1 of 1

Chromatogram and Results

Injection Details

Injection Name:	**P17 2nm RT: 15min**	*Run Time (min):*	**17,00**
Vial Number:	**RA4**	*Injection Volume:*	**30,00**
Injection Type:	**Unknown**	*Channel:*	**Raytest_Gabi**
Calibration Level:		*Wavelength:*	**254**
Instrument Method:	**YR 5_95 ACN_H2O 2ml 15min**	*Bandwidth:*	**n.a.**
Processing Method:	**Martins Processing Method**	*Dilution Factor:*	**1,0000**
Injection Date/Time:	**27.Aug.24 08:31**	*Sample Weight:*	**1,0000**

Chromatogram

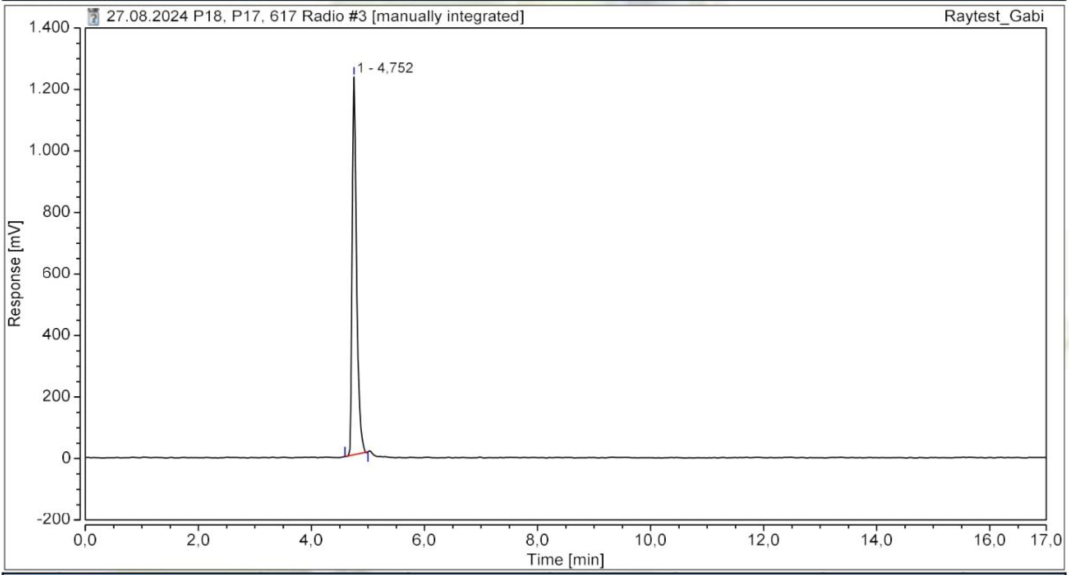

Integration Results

No.	Peak Name	Retention Time min	Area mV*min	Height mV	Relative Area %	Relative Height %	Amount
1		4,752	111,642	1228,262	100,00	100,00	n.a.
n.a.	Kiss 10	n.a.	n.a.	n.a.	n.a.	n.a.	n.a.
**Total:**			**111,642**	**1228,262**	**100,00**	**100,00**	

Default/Integration

© 2009-2020 Thermo Fisher Scientific Inc. All rights reserved.
Chromeleon 7.3.0.60919

Figure S22: Radio-HPLC of [^{177}Lu]Lu-P17.

Classification: Restricted

Chromatogram and Results

Injection Details

Injection Name:	**P18 2nm RT: 15min**	*Run Time (min):*	**17,00**
Vial Number:	**RA3**	*Injection Volume:*	**30,00**
Injection Type:	**Unknown**	*Channel:*	**Raytest_Gabi**
Calibration Level:		*Wavelength:*	**254**
Instrument Method:	**YR 5_95 ACN_H2O 2ml 15min**	*Bandwidth:*	**n.a.**
Processing Method:	**Martins Processing Method**	*Dilution Factor:*	**1,0000**
Injection Date/Time:	**27.Aug.24 08:12**	*Sample Weight:*	**1,0000**

Chromatogram

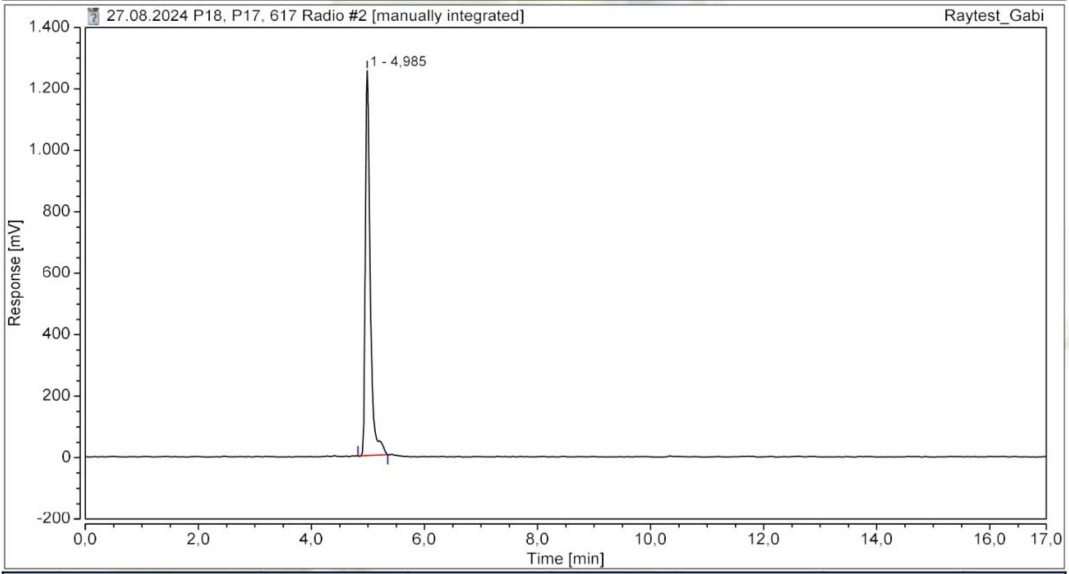

Integration Results

No.	Peak Name	Retention Time min	Area mV*min	Height mV	Relative Area %	Relative Height %	Amount
1		4,985	123,763	1252,822	100,00	100,00	n.a.
n.a.	Kiss 10	n.a.	n.a.	n.a.	n.a.	n.a.	n.a.
**Total:**			**123,763**	**1252,822**	**100,00**	**100,00**	

© 2009-2020 Thermo Fisher Scientific Inc. All rights reserved.
Chromeleon 7.3.0.60919

Figure S23: Radio-HPLC of [^{177}Lu]Lu-P18.

Classification: Restricted

Chromatogram and Results

Injection Details

Injection Name:	**617 2nm RT: 15min**	*Run Time (min):*	**17,00**
Vial Number:	**RA5**	*Injection Volume:*	**30,00**
Injection Type:	**Unknown**	*Channel:*	**Raytest_Gabi**
Calibration Level:		*Wavelength:*	**254**
Instrument Method:	**YR 5_95 ACN_H2O 2ml 15min**	*Bandwidth:*	**n.a.**
Processing Method:	**Martins Processing Method**	*Dilution Factor:*	**1,0000**
Injection Date/Time:	**27.Aug.24 08:49**	*Sample Weight:*	**1,0000**

Chromatogram

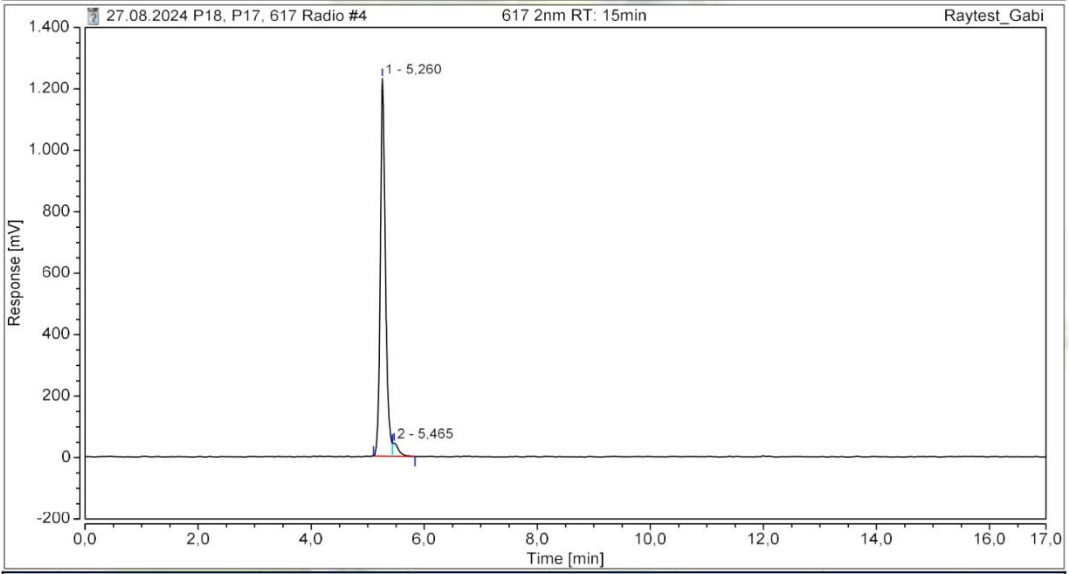

Integration Results

No.	Peak Name	Retention Time min	Area mV*min	Height mV	Relative Area %	Relative Height %	Amount
1		5,260	129,353	1229,827	96,01	96,59	n.a.
2		5,465	5,374	43,417	3,99	3,41	n.a.
n.a.	Kiss 10	n.a.	n.a.	n.a.	n.a.	n.a.	n.a.
**Total:**			**134,727**	**1273,244**	**100,00**	**100,00**	

Default/Integration

© 2009-2020 Thermo Fisher Scientific Inc. All rights reserved.
Chromeleon 7.3.0.60919

Figure S24: Radio-HPLC of [^{177}Lu]Lu-PSMA-617.

Classification: Restricted

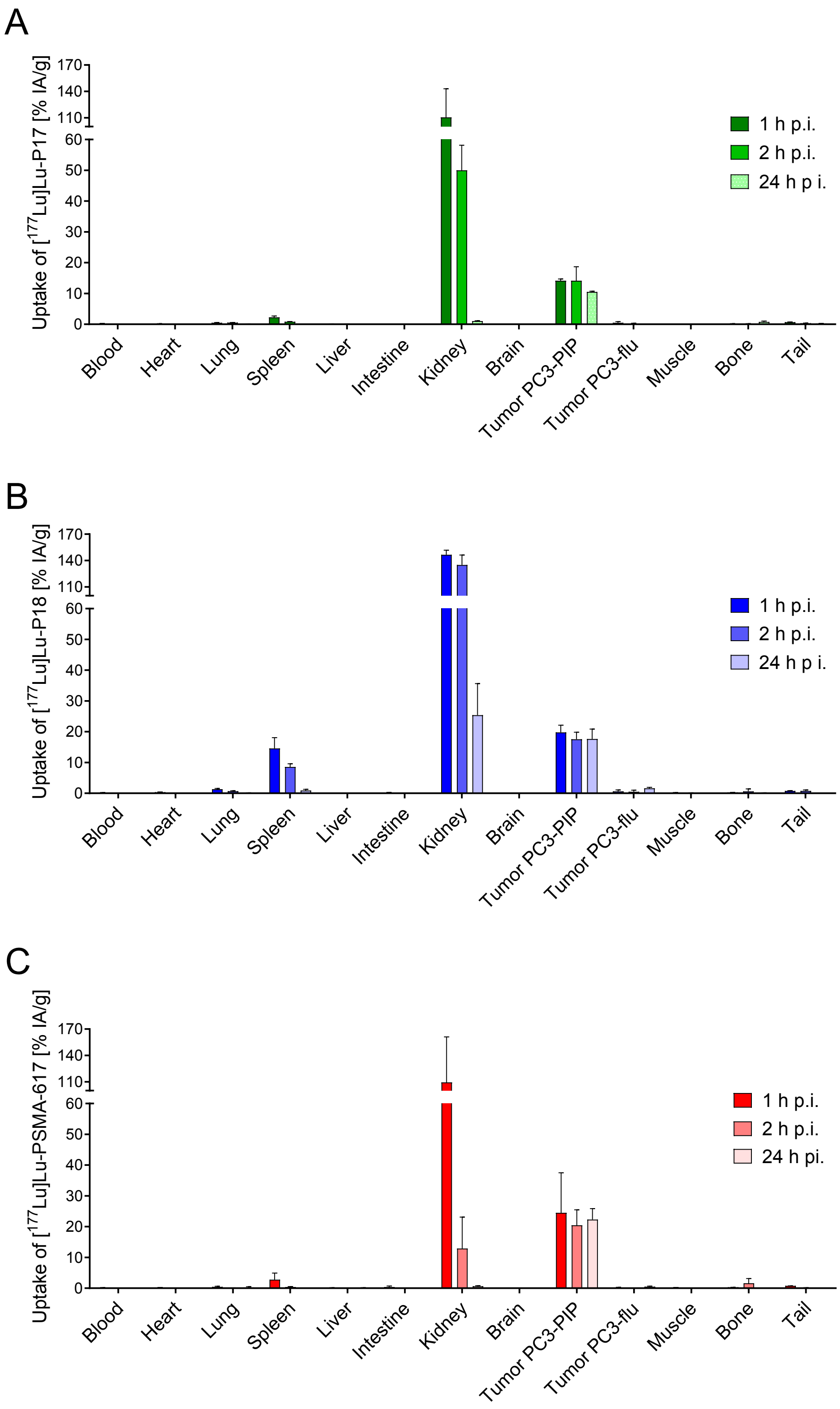

Figure S25: Uptake of radiolabeled PSMA ligands in PC3-PIP and PC3-flu tumors and various other tissues and organs at 1, 2, and 24 h p.i. for (A) [^{177}Lu]Lu-P17, (B) [^{177}Lu]Lu-P18, and (C) [^{177}Lu]Lu-PSMA-617. Data are average % IA/g ± SD (n = 3).

Classification: Restricted

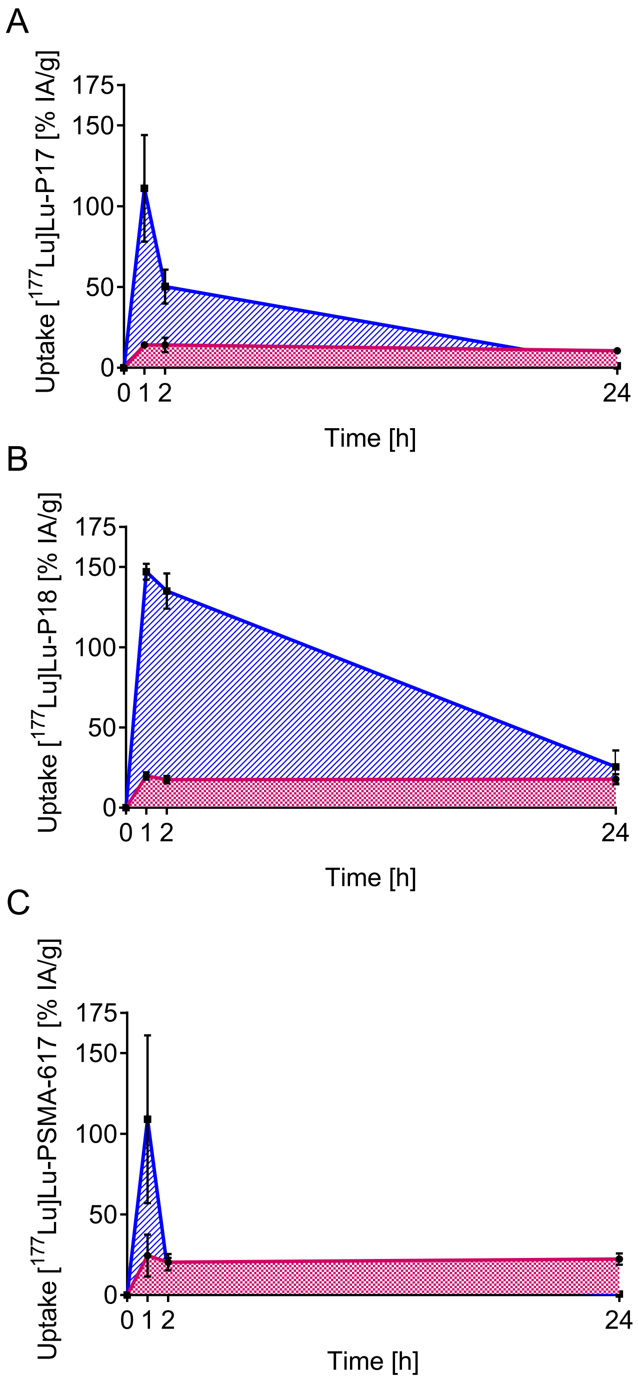

Figure S26: Uptake of radiolabeled PSMA ligands in PC3-PIP and kidneys depicted as area under the curve (AUC) values at 1, 2, and 24 h p.i. for (A) [^{177}Lu]Lu-P17, (B) [^{177}Lu]Lu-P18, and (C) [^{177}Lu]Lu-PSMA-617. Data are average % IA/g ± SD (n = 3).

Classification: Restricted

Table S1: Tumor-to-organ ratios from biodistribution data obtained in PC3-PIP and PC3-flu tumor-bearing balb/c nude mice at 1, 2, and 24 h p.i. of [^{177}Lu]Lu-P17, [^{177}Lu]Lu-P18, and [^{177}Lu]Lu-PSMA-617

	**1 h p.i. [^{177}Lu]Lu-P17**	**2 h p.i. [^{177}Lu]Lu-P17**	**24 h p.i. [^{177}Lu]Lu-P17**
**PC3-PIP-to-blood**	78.3	161	619
**PC3-PIP-to-muscle**	139	268	1620
**PC3-PIP-to-spleen**	6.3	17.5	87.9
**PC3-PIP-to-kidney**	0.13	0.28	9.8
**PC3-PIP-to-PC3-flu**	28.1	81.8	67.5
	**1 h p.i. [^{177}Lu]Lu-P18**	**2 h p.i. [^{177}Lu]Lu-P18**	**24 h p.i. [^{177}Lu]Lu-P18**
**PC3-PIP-to-blood**	106	276	2351
**PC3-PIP-to-muscle**	103	124	739
**PC3-PIP-to-spleen**	1.4	2.1	19.9
**PC3-PIP-to-kidney**	0.14	0.13	0.7
**PC3-PIP-to-PC3-flu**	31.9	36.1	10.8
	**1 h p.i. [^{177}Lu]Lu-PSMA-617**	**2 h p.i. [^{177}Lu]Lu-PSMA-617**	**24 h p.i. [^{177}Lu]Lu-PSMA-617**
**PC3-PIP-to-blood**	130	620	1306
**PC3-PIP-to-muscle**	168	971	5846
**PC3-PIP-to-spleen**	8.9	54.2	201
**PC3-PIP-to-kidney**	0.22	1.6	35.1
**PC3-PIP-to-PC3-flu**	144	447	43.6

Data are averages (n = 3).

Classification: Restricted

Adjusted methods for the preparation of resin-immobilized Glu-urea-Lys for P17 and P18

Scheme S7: Conversion of H-Glu(O*t*Bu)-O*t*Bu to OCN-Glu(O*t*Bu)-O*t*Bu for further conjugation with resin-immobilized lysine.

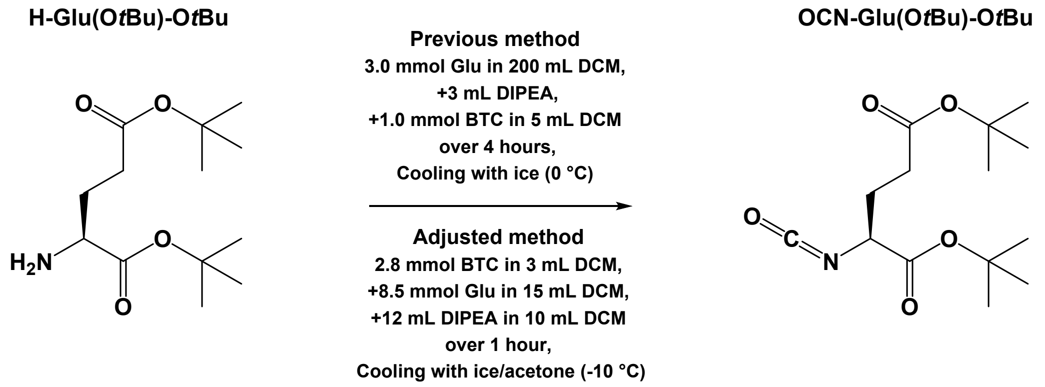

Scheme S8: Conversion of H-Glu(O*t*Bu)-O*t*Bu to CDI-adduct-Glu(O*t*Bu)-O*t*Bu for further conjugation with resin-immobilized lysine.

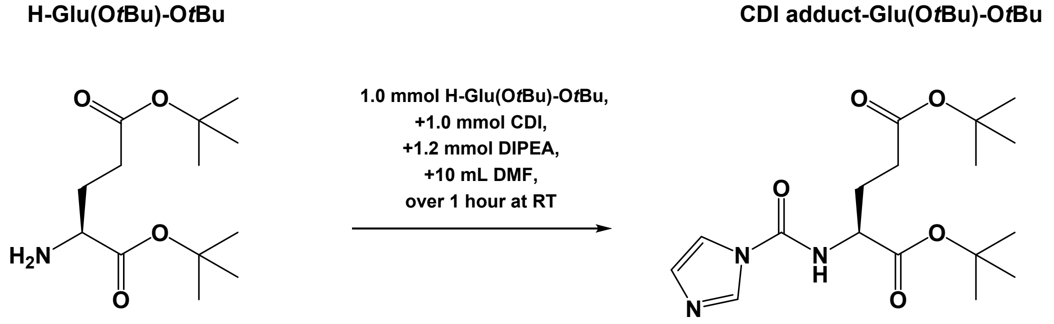

Classification: Restricted
